# Focusing on CD8^+^ T-cell phenotypes: improving solid tumor therapy

**DOI:** 10.1186/s13046-024-03195-5

**Published:** 2024-09-28

**Authors:** Zhouchi Yao, Yayun Zeng, Cheng Liu, Huimin Jin, Hong Wang, Yue Zhang, Chengming Ding, Guodong Chen, Daichao Wu

**Affiliations:** 1 Department of Hepatopancreatobiliary Surgery, The First Affiliated Hospital, Laboratory of Structural Immunology, Hengyang Medical School, University of South China, Hengyang, Hunan 421001 China; 2 Department of Histology and Embryology, Hengyang Medical School, University of South China, Hengyang, Hunan 421001 China; 3 Department of Scientific Research, The First Affiliated Hospital of Jinzhou Medical University, Jinzhou, Liaoning 121001 China

**Keywords:** Immunotherapy, CD8^+^ T cell, Solid tumor, Engineered T cell receptor -T cells, Immune checkpoint, T cell redirection, Gene editing, Immune reprogramming, Metabolic reprogramming

## Abstract

Vigorous CD8^+^ T cells play a crucial role in recognizing tumor cells and combating solid tumors. How T cells efficiently recognize and target tumor antigens, and how they maintain the activity in the “rejection” of solid tumor microenvironment, are major concerns. Recent advances in understanding of the immunological trajectory and lifespan of CD8^+^ T cells have provided guidance for the design of more optimal anti-tumor immunotherapy regimens. Here, we review the newly discovered methods to enhance the function of CD8^+^ T cells against solid tumors, focusing on optimizing T cell receptor (TCR) expression, improving antigen recognition by engineered T cells, enhancing signal transduction of the TCR-CD3 complex, inducing the homing of polyclonal functional T cells to tumors, reversing T cell exhaustion under chronic antigen stimulation, and reprogramming the energy and metabolic pathways of T cells. We also discuss how to participate in the epigenetic changes of CD8^+^ T cells to regulate two key indicators of anti-tumor responses, namely effectiveness and persistence.

## Introduction

The rapid development of cancer immunotherapy has proven to be effective in restoring T cell-mediated immune responses [1, 2]. The combined application of immune checkpoint blockade and adoptive cell therapy has not only achieved unprecedented clinical responses in patients with refractory solid tumors but also brought long-term clinical remission to patients who were considered incurable due to tumor metastasis [3–5]. The cornerstone of immunotherapy is Cluster of differentiation 8^+^ (CD8^+^) cytotoxic T lymphocytes, which can directly destroy cancer cells [6]. Therefore, promoting CD8^+^ T cell-specific immune responses is considered as the focus of current cancer immunotherapy, and effector CD8^+^ T cells (T_eff_) are the preferred immune cells against tumors.

Naive CD8^+^ T cells, stimulated by tumor-associated antigens presented by the major histocompatibility complex (MHC) on the surface of tumor cells, are promoted to differentiate into Teff under the assistance of co-stimulatory molecules [7]. This process is accompanied by transcriptional, epigenetic, and metabolic reprogramming, as well as the acquisition of characteristic effector features, such as the ability to produce cytokines and cytotoxic molecules [8]. However, persistent tumor antigen stimulation can lead to the transition of CD8^+^ T_eff_ to exhausted CD8^+^ T cells (T_ex_). T cell exhaustion was first characterized in lymphocytic choriomeningitis virus (LCMV)-specific T cell receptor (TCR) transgenic CD8^+^ T cells during chronic infection [9]. The characteristics of T_ex_ in tumors primarily include the loss of effector function, persistent and elevated expression of inhibitory receptors (IRs), alterations in epigenetic and transcriptional profiles, and unique metabolic pathways [10, 11]. In most solid tumors, various reasons lead to a low burden of intracellular mutant antigens or down-regulation of MHC molecule expression in tumor cells, resulting in tumor cells being insufficient to be recognized by TCRs or unable to trigger the expansion and functional activation of CD8^+^ T_eff_ after TCRs recognition [12–14]. The complex immune microenvironment of solid tumors hinders the high-throughput infiltration of CD8^+^ T_eff_, furthermore, the crosstalk between tumor cells, tumor matrix, and myeloid-derived suppressor cell populations (MDSCs) in the tumor microenvironment (TME) inhibits TCR signal transmission and limits T_eff_ function [15]. Meanwhile, nutritional stress and metabolic waste accumulation caused by cancer cells [16] also induce energy and biosynthetic metabolic disorders, as well as alterations in transcriptome and epigenetic characteristics, thereby accelerating the exhaustion process of CD8^+^ T_eff_ cells [17, 18].

With the rapid advancements in the fields of antigen identification, T cell biology, and gene therapy, a variety of anti-tumor immunotherapeutic strategies based on T cells have been proposed to enhance T cell function and redirect tumor-specific antigens [19]. The application of adoptive cell therapy, which involves the ex vivo expansion of tumor-infiltrating CD8^+^ T lymphocytes from patients or CD8^+^ T lymphocytes from donors stimulated by specific tumor antigens to sufficient numbers, and then reinfusion into patients who have undergone lymphocyte chemotherapy, has largely compensated for the low immunogenicity of “cold tumors” and the immune blockade of the suppressive tumor microenvironment [20–22]. The application of genetic engineering in adoptive cell therapy (ACT) has accelerated the progress of therapies involving chimeric antigen receptor (CAR) and engineered T cell receptor (TCR)-T cells. The antigen recognition of CAR-T relies on the single-chain variable fragment (ScFv) on the engineered receptor binding to the antigen on the membrane surface [23]. This presents challenges as it may not effectively recognize intracellular tumor antigens and cannot avoid the recognition of somatic common antigens, potentially leading to a low anti-tumor immune response and high off-target cytotoxicity [24, 25].TCR-T, which lyse tumor cells through exogenous affinity-matured TCRs, strictly recognizes antigen epitopes presented by MHC-I class molecules, making low mutation load neoantigens within tumor cells effective targets [26]. Importantly, a series of neoantigens have been identified, and multiple clinical trials have demonstrated the efficacy of TCR-T cell therapy against solid tumors [27–29].In addition to cell therapy, the application of engineered TCRs in T cell mobilization molecules, as well as the design of TCR mimic antibodies (TCRm Abs) based on the trajectory of TCR recognition of peptide-MHC (p-MHC) complexes, all contribute uniquely to the redirection of T_eff_ in solid tumors [30–32]. In this review, we focus on the phenotypes of CD8^+^ T cells, discussing numerous feasible strategies to enhance CD8^+^ T cell therapy for solid tumors in the process of the initial expression and assembly of TCR to the activation and exhaustion of T cells, from the perspectives of T cell structure and genetic engineering modifications, and immune and metabolic program regulation.

## Engineering of CD8+ T cells potentiate the anti-tumor efficacy

### T cell receptor (TCR) engineered T cell therapy

The interaction between T cell receptors (TCRs) and their homologous tumor antigens is the core of anti-tumor immune responses. However, the inevitable heterogeneity of immune composition and phenotype spectrum within tumor-infiltrating lymphocytes [33–35] leads to low reactivity and variability in the intrinsic ability of TCRs to recognize autologous cancers [36, 37]. Neoantigen-specific TCRs (NeoTCR), due to their central tolerance, exhibit a higher affinity for mutated p-MHC with stronger interaction and stability [38, 39], and their ability to induce tumor regression has been characterized in various refractory solid tumors originating from different types of somatic cell mutations [27, 29, 31, 40]. Understanding the structural details of the interaction between TCR and its homologous p-MHCs provides a theoretical basis for exploring the structure-function relationship of neoantigen-reactive TCRs [41, 42]. NeoTCR-T cell therapy is currently a key area of exploration in immunotherapy. The development of individual cancer epitope identification technology has overcome the bias caused by the diversity of human leukocyte antigen (HLA) alleles and the huge spatial potential of peptide epitopes [43, 44]. The combination of isolating the original paired TCRαβ chains from polyclonal T cells and TCR response analysis based on next-generation sequencing (NGS) provides opportunities for high-throughput screening of endogenous reactive TCRs [45–47] but lacks control over the accuracy and frequency of individual TCRs in the library. The “sequence-and-synthesize” based ex vivo TCR gene editing [48, 49] and the “T-FINDER” [50], that combines neoantigen epitope identification and specific TCR screening, have made good supplements to this. Although the tumor neoantigen mutation burden (TMB) of individuals is directly related to the reactivity of NeoTCR-T cells [51], the heterogeneity of TMB emphasizes the importance of increasing the stability, affinity, and persistent functional activity of adoptively transferred NeoTCR-T cells in enhancing ACT [52].

#### Enhancing correct TCR membrane expression in engineered T cells

**Fig. 1:**
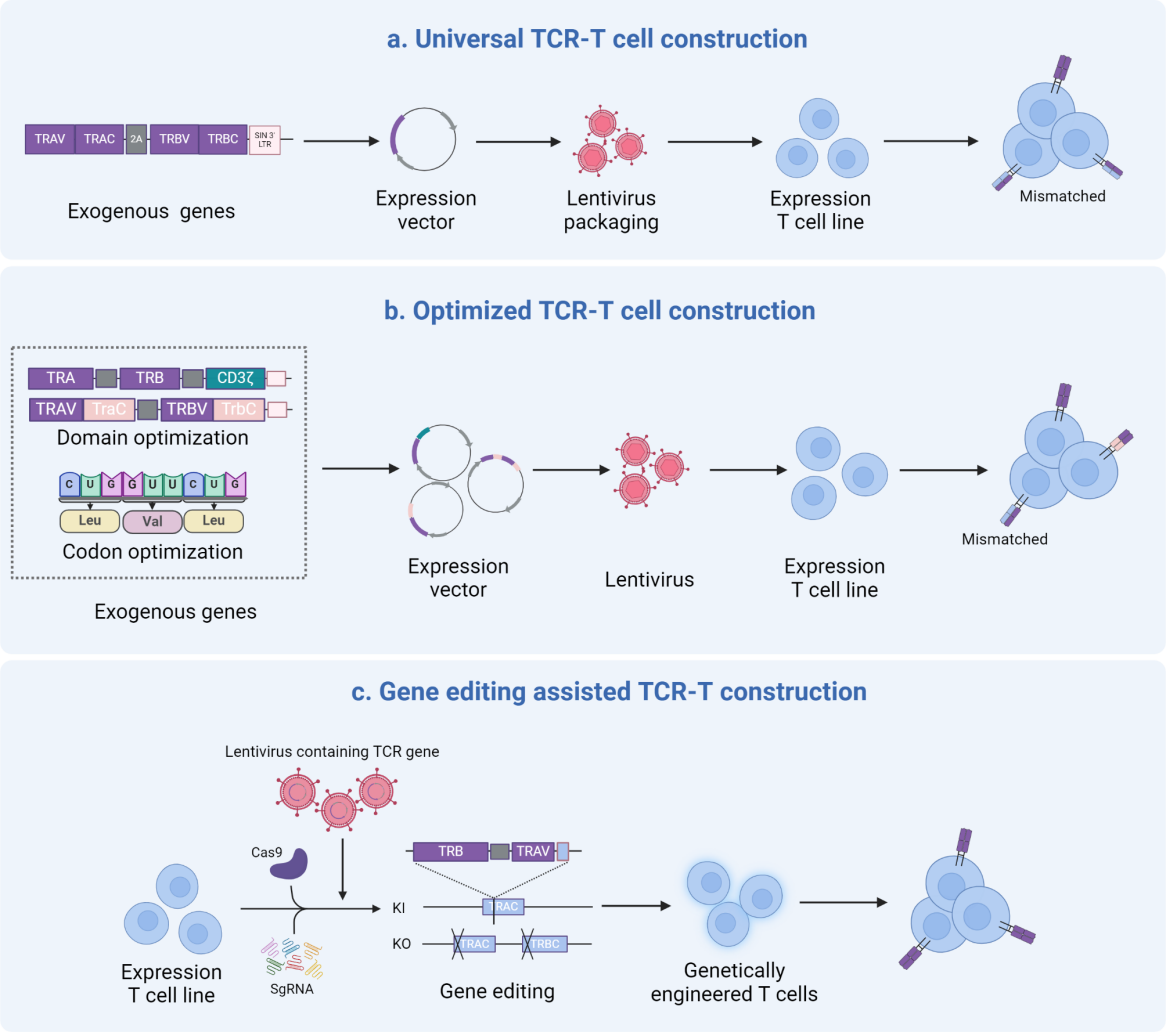
Enhancement of Neoantigen-Specific Engineered TCR Expression Efficiency. (**a**) CD8^+^ T cells are transduced with neoantigen-specific engineered TCRs via lentivirus. Both endogenous and engineered TCRs are expressed on the cell surface, with a potential for mismatched TCR expression. (**b**) TCR domain engineering strategies are employed to reduce mismatched TCR expression. These strategies include the introduction of murine TCR constant domains, replacement of TCRαβ chain constant domains, or co-expression of TCRαβ chains with CD3ζ. Additionally, amino acid modifications such as the introduction of optimal amino acids, additional disulfide bonds, elimination of conserved N-glycosylation sites, increased hydrophobicity of the TCR transmembrane domain, and substitution of specific structural anchor amino acids in TCRαβ enhance engineered TCR expression. (**c**) CRISPR-Cas9 base editing is utilized to inhibit endogenous TCR expression, thereby reducing mismatched TCRs. CRISPR-Cas9 gene editing knocks out endogenous TCR genes and precisely integrates engineered TCR genes to promote their expression

The primary success of tumor antigen-specific TCRs adoptive cell therapy is achieved through the transition to physiological T cells [53]. Previously, the transfer of exogenous TCR genes was primarily mediated by viruses [54], then semi-randomly expressed in engineered T cells, and assembled on the cell membrane with the CD3 signaling apparatus into an octameric TCR-CD3 complex with a 1:1:1:1 stoichiometry of TCRαβ:CD3γε:CD3δε:CD3ζζ [55]. A higher density of TCRs on the cell surface is believed to facilitate reaching the T cell activation threshold more easily [56]. However, due to the presence of endogenous TCRs, the expression of transgenic TCRs is often suppressed [57], or erroneous TCRαβ pairing occurs, causing engineered T cells to produce new antigenic properties, which may trigger auto-reactivity [58] (Fig. 1a). Therefore, ideally, successful TCR-T cells require efficient expression of the required αβ chains and are subject to physiological regulation, which is a major challenge in the field of TCR engineering.

For this reason, many biological modification methods applied to the TCRαβ chain structural domain have been attempted (Fig. 1b). Since the mouse TCR constant domain (C) α/β preferentially pairs, replacing its amino acid residues with the human TCR Cα/Cβ sequence can reduce the mismatch of human transgenic TCR [59], and minimally murinized TCR mutants also support the preferential pairing of transferred TCR chains [60]. Although murinized TCR chains may introduce potentially immunogenic exogenous sequences, no correlation has been found between immunogenicity and T cell functionality and clinical outcomes in patients receiving such engineered T cell therapy [61]. By swapping the therapeutic TCR Cα/Cβ structural domains, domain-swapped TCRs can assemble with CD3 and express on the T cell surface, reducing mismatches while excluding heterologous sequences in cell products [62]. A 3-domain single-chain TCRs framework [63] that depends on co-expression of the TCRα constant region can prevent unnecessary TCRαβ pairing and maintain a structure and functionality similar to the fully assembled double-chain TCR/CD3 complex [64]. In addition, co-transduction of TCR and CD3ζ increases the surface expression of the introduced TCR and reduces the mismatch with endogenous TCR chains [65], and its genetic tandem with auxiliary or co-stimulatory molecules can also enhance cell co-stimulation [66].

In addition to overall domain modifications, residue modifications between receptor chains are also expected to promote stable TCR expression. In this regard, various strategies have been designed and functionally validated, including optimal amino acid substitutions within the TCR framework region [67], the introduction of additional disulfide bonds at the TCR Cα/Cβ interface [68], selective removal of conservative N-glycosylation sites [69], and selective increase of TCRαβ Transmembrane domain (TMD) hydrophobicity [70]. Furthermore, swapping amino acid residues at the TCR Cα/Cβ interface that are linked to “knob-into-hole” structural interactions and electrostatic environments can also promote selective pairing of introduced TCRs [71]. Simultaneously, synonymous codon substitutions in the TCR coding sequence, as well as the omission of messenger ribonucleic acid (mRNA) instability motifs and implicit splice donor sites, allow for efficient expression of transgenic TCRs [72].

These efforts have indeed increased the cell membrane expression of transgenic TCRs, but mismatches cannot be eliminated. With the deep exploration of the field of genome editing, it has been found that actively knocking out endogenous TCR genes in engineered T cells fundamentally aids the efficient and stable expression of transgenic TCRs [73] (Fig. 1c). Initially, zinc-finger nucleases (ZFNs) [74] and transcription activator-like effector endonucleases (TALENs) [75] were used to disrupt endogenous TRAC and TRBC1/2 sites in engineered T cells, and subsequently emerged clustered regularly interspaced short palindromic repeats (CRISPR)– CRISPR-associated protein 9 (Cas9) [76] completed this work more efficiently [77]. However, these strategies induce double-strand DNA break behavior, which may lead to off-target genomic effects [78]. To change this situation, a CRISPR-based DNA base editor has been developed, which does not disrupt protein expression by inducing DNA double-strand breaks [79], and its application at TRAC, TRBC1/2 sites may provide a safer choice for genetically deleting endogenous TCR [80, 81]. In addition, CRISPR-Cas9 precise genome editing that does not rely on viral vectors can target the integration of NeoTCRs genes at the original TRAC site while knocking out endogenous TCR [29, 82, 83]. Gene-targeted integration is achieved through co-electroporation of human primary T cells, CRISPR-Cas9 ribonucleoprotein (RNP) complexes, and homologous double-stranded DNA repair templates. Compared with the semi-random insertion of NeoTCR genes by viral transduction, targeted integration allows NeoTCR genes to be physiologically regulated by endogenous promoters, providing engineered T cells with enhanced functionality and predictable safety, and reducing immune exhaustion [73]. Although the efficiency of targeted integration is lower than viral transduction, various improvement methods, such as single-stranded DNA (ssDNA) homology-directed repair (HDR) templates [84], nanoplasmid [85], stabilization of Cas9 RNP sand enhancement of template nuclear shuttling [86], have been designed to improve editing efficiency.

#### Increasing TCR effective recognition of antigen

The activation, proliferation, infiltration, and effector function of CD8^+^ T cells, as well as the duration of T cell responses, partly depend on the recognition strength of TCR to its homologous p-MHC complexes [87, 88]. This recognition strength is determined by the structural affinity based on the interaction of TCR with p-MHC complexes [89], which can be characterized by the dissociation kinetics of TCR with monomeric p-MHC complexes [90]. The non-covalent binding between different residues in TCR and p-MHC complexes reflects the contribution of this site to TCR antigen recognition. By analyzing the shared base sequence of TCR recognition-related core sequence, key conservative residues driving TCR recognition can be found, providing insights for analyzing the potential features of TCR epitope specificity spectrum [91]. The complementary determining regions (CDRs) in the variable structural domain of TCRαβ are crucial in the interaction with p-MHC complexes due to their antigen recognition and high variability. Usually, the CDR1 and CDR2 loops encoded by the same lineage gene have conservative interactions with MHC molecules, while the CDR3 loop is highly variable and mainly responsible for antigen peptide recognition [92]. The original structure of TCR can be artificially modified. The exploration of CDRs affinity maturation has been ongoing, and related work is dedicated to mutating CDRs residues to enhance TCR affinity and determine its basic characteristics and core base sequence [93, 94]. Mutation of a single CDR3 loop [95] or combined CDRs loops [96] to increase TCR affinity is a mature strategy, theoretically leading to up to 10^7^ times higher affinity than the natural sequence [93], and its safety and effectiveness have been confirmed in subsequent clinical trials. In addition to CDRs directed evolution, the TCR affinity engineering platform constructed by ex vivo antigen-driven progenitor T cells has also been verified [97]. Changes in CDRs sequence may also change TCR antigen specificity, bringing antigen cross-reactivity while increasing affinity, which may cause fatal off-tumor or off-target toxicity [98, 99]. The combination of structure-guided positive and negative design, to fine-tune TCR in a way that reduces cross-reaction but keeps affinity close to the optimal level [100], seeks to redistribute attractive interactions on the TCR-p-MHC interface, thereby reducing the recognition of off-target peptides [101]. For TCR-p-MHC without readily available crystal structure, deep learning algorithms have been used for three-dimensional predictive analysis to train TCR’s binding ability to p-MHC complexes [102]. AlphaFold, a generative AI model using neural network architecture, can be applied to the development of a generalizable prediction model for TCR and p-MHC complexes specific binding [103]. With the iterative evolution of the model, the third-generation AlphaFold predicts the structure of TCR and p-MHC complexes interaction with unprecedented accuracy [104].

**Fig. 2:**
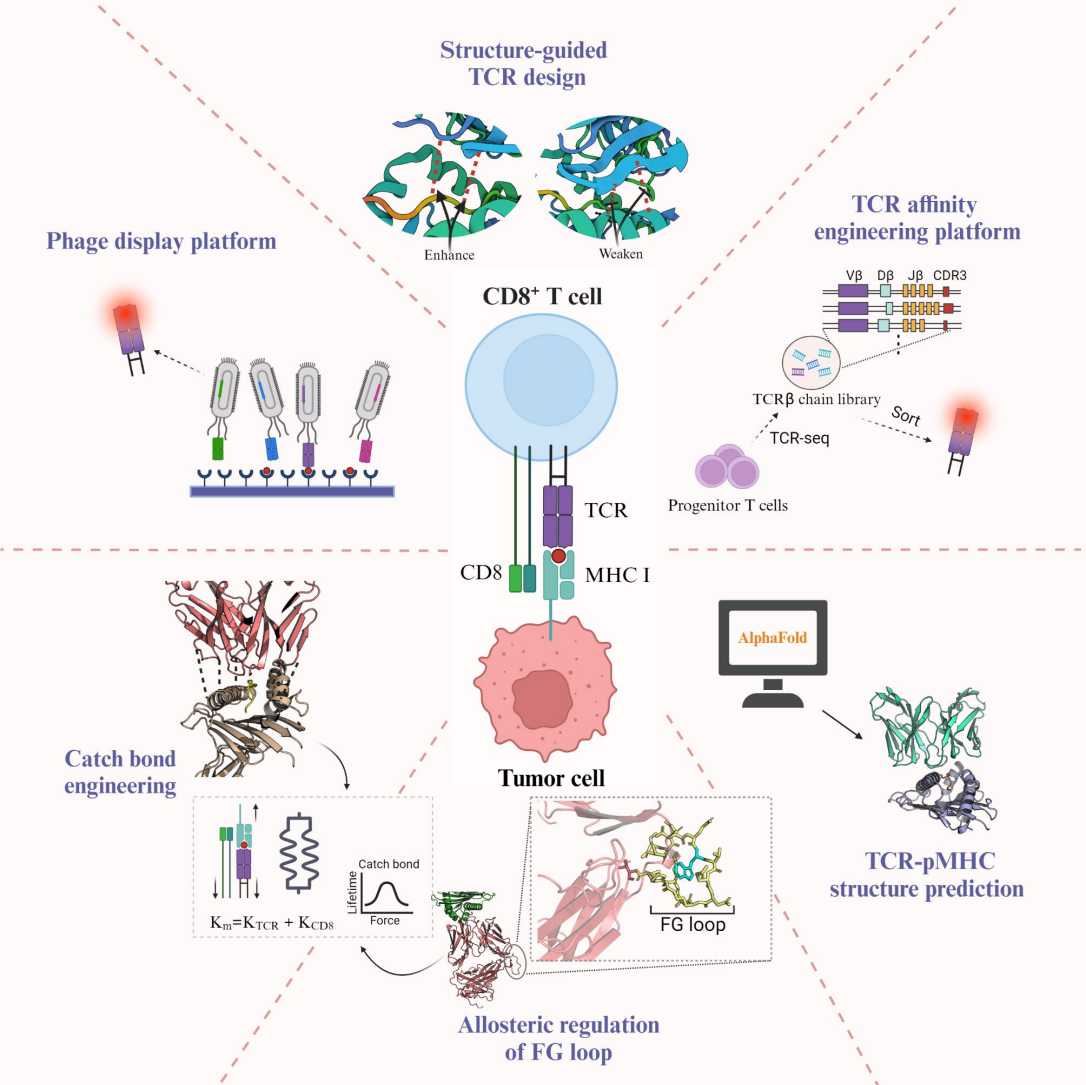
Enhanced and Stabilized Neoantigen Recognition of TCR. Phage display platforms can be used to screen for mature, antigen-specific TCRs with high affinity, but there may be unknown reactivities. Structure-guided positive and negative selection of TCR design, which increases the connection between TCR and antigen epitopes while reducing the interaction between TCR and MHC, can significantly reduce cross-reactivity. Using a TCR affinity engineering platform built on progenitor T cells, it is possible to screen for neoantigen-reactive TCRs with increased affinity and low cross-reactivity. Using an iteratively evolved protein structure prediction model based on deep learning algorithms, it is possible to accurately predict the structure of non-readymade TCR-p-MHC complexes, guiding the analysis of neoantigen-specific TCRs. Based on the mechanical model of TCR-p-MHC interaction, stabilizing the FG loop structure of the TCRβ constant domain and introducing intermolecular forces at non-direct contact sites of the TCR and p-MHC complexes can induce the formation of catch bonds and extend their lifespan, enhancing TCR recognition of p-MHC complexes without significantly changing affinity

Mechanical transmission can enhance TCR’s recognition of p-MHC complexes. After TCR binds to the ligand, T cells change the TCR-p-MHC bond lifetime by applying endogenous forces - excitatory forces promote the formation of “catch bonds” to extend bond life, antagonistic forces promote the formation of “slip bonds” to shorten bond life [105]. More stable ligand recognition induces the formation of longer-lasting TCR-pMHC-CD8 three-molecule “catch bonds” [106, 107], which is a trans (lateral) heterodimer formed by the co-receptor CD8 interacting with the extracellular p-MHC complexes and the intracellular TCR-CD3 complexes and coupled by related kinases, which can transmit signals from the inside out [108]. It has been found that the FG loop conformation of the TCRβ chain constant structural domain controls the catch bond life of the variable structural domain module and controls peptide recognition through force-driven conformational changes [109]. Therefore, understanding the internal mechanism of TCR mechanosensing can explain why and how force enhances TCR antigen recognition and signal transduction without changing structural affinity [110]. Excitingly, the biophysics-based “catch bond” engineering that has emerged in response can increase TCR sensitivity while reducing potential adverse cross-reactivity. A feasible strategy is to introduce polar or charged amino acids into the CDRs residues that do not directly contact TCR and p-MHC complexes, to increase the molecular resistance of hydrogen bonds or salt bridges faced during the dissociation process of TCR-p-MHC trimer, thereby maintaining stable antigen epitope recognition and binding of p-MHC complexes [111] (Fig. 2).

### TCRmimic-engineered TCR-T cells

**Fig. 3:**
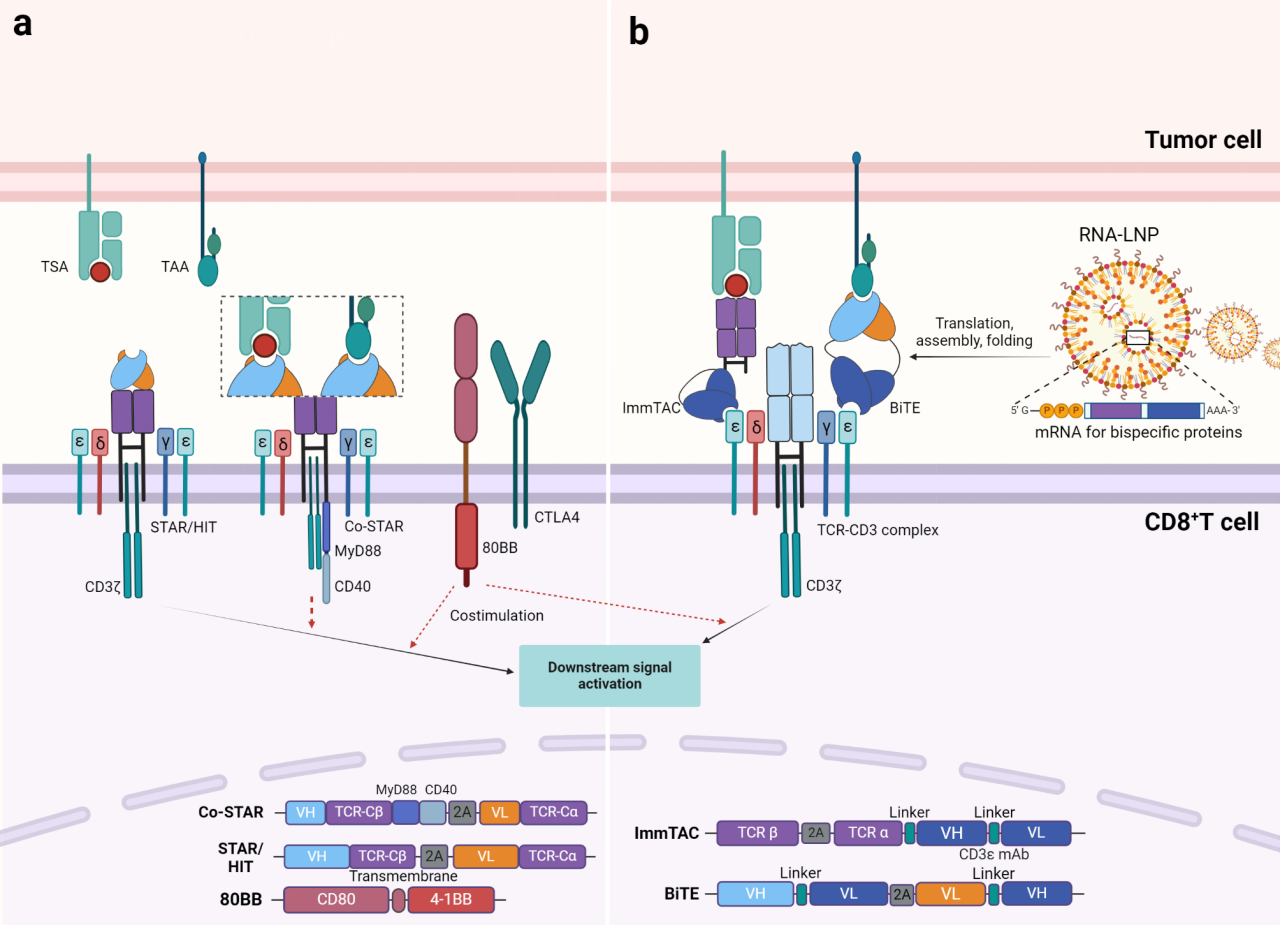
Affinity-enhanced TCR-mimic engineered T cells and bispecific protein molecules. **a**. The Synthetic TCR antigen receptor (STAR)/HLA-independent TCR (HIT), formed by replacing the physiological TCR variable domains with those of TCRm Abs or monoclonal antibodies (mAbs), recognize tumor-specific antigens (TSA) or tumor-associated antigens (TAA) and induce proximal phosphorylation signaling of the receptor-CD3 complex. The fusion protein 80BB, composed of the extracellular domain and transmembrane domain of CD80, and intracellular domain of 4-1BB, can convert the inhibitory signal from CTLA4 into a co-stimulatory signal, promoting proximal TCR signal transduction. The Myd88-cd40 co-stimulation module fused to the STARβ chain significantly enhances the antigen sensitivity of T cells. The bottom of the figure shows a schematic diagram of the gene arrangement of Co-STAR, STAR/HIT and 80BB, which are arranged in the order of proteins domains from the N-terminus to the C-terminus. Different chains are linked by the self-cleaving peptide sequence P2A. **b**. The immune-mobilizing monoclonal TCR against cancer (ImmTAC), composed of the soluble TCR targeting tumor-specific antigens and the single-chain fragment variable (ScFv) composed of the light and heavy chain variable structural domains of monoclonal antibodies targeting CD3ε, as well as the bispecific T cell engager (BiTE), composed of TCRm Abs or mAbs targeting tumor antigens and ScFv targeting CD3ε, induce polyclonal T cells to redirect against tumor cells and activate proximal TCR signals in a CD3-dependent manner. Bispecific protein molecules can be stably delivered to the tumor interior in the form of mRNA by lipid nanoparticle carriers (LNP). The bottom of the figure shows a schematic diagram of the gene arrangement of ImmTAC and BiTE, which are arranged in the order of proteins domains from the N-terminus to the C-terminus. Different chains are linked by the sequence of self-cleaving peptide P2A. Different domains within the same chain are connected by sequence of flexible linkers

Synthetic TCR antigen receptor (STAR)/HLA-independent TCR (HIT) is a newly engineered receptor generated by replacing the variable domains of TCRα and β chains with the heavy and light chain variable domains of antibodies while retaining the complete TCR constant structural domain [112, 113]. Since only the variable domain of the receptor is changed, STAR/HIT can still form a complete TCR signal transmission module in conjunction with the CD3 complexes, and accept physiological regulation consistent with TCR. Compared to TCR-T and CAR-T, STAR/HIT-T cells, endowed with higher antigen affinity and superior tumor infiltration capabilities, demonstrate stronger antigen sensitivity and more stable cellular functions, enabling them to control, and even eradicate, tumor growth in various murine solid tumor models [112, 113], thereby providing a novel framework for the optimized design of engineered T cells. Co-stimulatory signals are a key factor in enhancing TCR signals. A switch fusion receptor called 80BB, composed of the extracellular structural domain of CD80 and the cytoplasmic structural domain of 41-BB, can transform signals from inhibitory receptors into co-stimulatory signals, enhance and maintain CD3-dependent T cell responses, and thereby improve the anti-tumor ability of STAR, HIT, or TCR-engineered T cells [114] (Fig. 3a). By concatenating modules that drive the nuclear factor (NF)-κB signal, namely MyD88 and CD40, to the C-terminus of the STAR β chain, a novel co-stimulation model is established, which gives rise to a variant of Co-STAR-T cells that not only exhibit enhanced sensitivity to antigens but also possess an improved ability to home to tumors over an extended period of time, thereby delivering superior anti-tumor efficacy, and paving the way for a new approach in T cell therapy specifically tailored for tumors characterized by an extremely low antigen density [115].

## Redirection of T cells potentiate the anti-tumor efficiency

### Immune-mobilizing monoclonal TCR against cancer (ImmTAC)

In most solid tumors, low immunogenicity and a highly suppressive tumor microenvironment limit the infiltration and activation of CD8^+^ T cells. At the same time, small soluble proteins that target both tumor cells and T cells can overcome the biophysical characteristics of tumors, utilize natural antigen presentation pathways and specific antigen recognition structures to target tumor cells, trigger the relocation of polyclonal CD8^+^ T cells in the interstitium, promote the formation of immune synapses, and ultimately transform the immune rejection phenotype of the tumor into an immune inflammation phenotype (Fig. 3b).

TCRαβ chains are recombinantly expressed as soluble single-chain heterodimers, which are then fused with a single-chain variable fragment (ScFv) derived from an anti-CD3ε antibody through a flexible linker to generate a bispecific protein that can simultaneously target polyclonal T cells and tumor cells expressing specific p-MHC complexes, known as ImmTAC [116]. In vitro, ImmTAC fused with a TCR that has increased affinity after CDRs mutation by biological display technology can mediate CD8^+^ T cells to show specific cell lysis to tumor cells with low antigen density [31, 116, 117]. Tebentafusp, which specifically recognizes HLA-A*02:01 complexed with the melanoma-associated antigen gp100, is currently the only approved ImmTAC molecule for the treatment of unresectable or metastatic uveal melanoma and other solid tumors [118]. Propelled by the success of tebentafusp, the phase I/II clinical trials of ImmTAC molecules, which target the HLA complexed with the tumor antigens melanoma-associated antigen A4 (MAGE-A4)、New York esophageal squamous cell carcinoma 1 (NY-ESO-1) and L antigen family member-1 A (LAGE-1 A), have been completed (NCT03973333、NCT03515551), and concurrently, ImmTAC molecule that targets the HLA complexed with the tumor antigen preferentially expressed antigen in melanoma (PRAME) is preparing to enter clinical trials (NCT04262466).

### Bispecific T cell engager (BiTE)

Another bispecific protein is the BiTE (Fig. 3b), which is composed of two ScFvs derived from antibodies - one recognizes the p-HLA epitope on the surface of tumor cells, and the other binds to the CD3 complexes on the surface of T cells, and is covalently connected by a peptide linker. It has achieved significant success in hematological malignancies [119, 120]. Compared to TCR, using ScFv as an antigen binding domain derived from TCR mimic Abs has the advantage that they are easy to improve in affinity and high-throughput synthesis by biological display technology [121]. With the advancement of high peptide specificity and MHC-restricted TCRm Abs screening and synthesis methods [30], a growing number of TCRm Abs targeting solid tumor neoantigens considered undrugneable and monoclonal Abs targeting solid tumor-associated antigens have been identified and used to synthesize BiTEs for anti-tumor immunity [122–124]. At present, a phase I clinical trial of a TCR-like antibody targeting MAGE-A4 in patients with recurrent or refractory (R/R) metastatic solid tumors is underway (NCT03132922) [125, 126]. Another delta-like ligand 3 (DLL3)/CD3 BiTE targeting DLL3, IMDELLTRA™ (tarlatamab-dlle), has completed phase I and II clinical trials (NCT03319940, NCT05060016) [127, 128], and has recently received accelerated approval from the U.S. Food and Drug Administration (FDA), becoming the first and only T cell engager for the treatment of extensive-stage Small-cell lung cancer (SCLC) [129]. Moreover, the tri-specific DLL3-targeting T-cell engager [130], which additionally targets the albumin binding domain to extend its half-life, is currently undergoing phase I / II clinical trials (NCT04471727) in patients with DLL3-associated cancers. Furthermore, bispecific T cell engagers targeting glypican-3 (GPC3), prostate-specific membrane antigen (PSMA) and claudin (CLDN) 18.2 are respectively undergoing phase I clinical trials in patients with hepatocellular carcinoma (HCC) (NCT05450562), metastatic castration-resistant prostate cancer (mCRPC) (NCT04740034) and pancreatic ductal adenocarcinoma (PDAC) and gastric and esophageal malignancies (NCT05164458, NCT05164458). Simultaneously, a bispecific T cell engager targeting the epithelial cell adhesion molecule (EpCAM) is in phase II clinical trials in patients with malignant ascites from epithelial cancers (NCT06266091). As well known, the tumor affinity of BiTE can be improved by changing the antibody structure to increase the number of binding elements targeting tumor cells. Cibisatamab, a bispecific T cell engager with two Fab structural domains recognizing carcinoembryonic antigen (CEA) and one Fab structural domain binding to CD3 [131], is currently undergoing a phase I clinical trial (NCT02324257) in patients with locally advanced or metastatic solid tumors expressing CEA.

Due to their relatively small molecular weight, ImmTAC and BiTE face a relatively high plasma clearance rate in therapeutic activities, leading to a short plasma half-life in patients, thereby preventing them from fully exerting their functions. In the past, a method of fusing small molecule proteins with Fc molecules modified by IgG to extend the half-life had been proposed [132]. With the development of nanocarrier technology, lipid nanoparticles (LNPs) with superior targeting and pharmacokinetics have been widely used for the in vivo delivery of mRNA vaccines [132]. Inspired by cancer mRNA vaccines, an mRNA (RiboMABs) containing 1-methyl pseudouridine for encoding His-tagged Bispecific T cell engager antibodies have been designed [133]. Recently, a clinical trial is underway to improve the efficacy of bispecific antibodies by applying BNT142, which is a lipid nanoparticle (LNP), formulated RiboMABs encoding claudin 6 (CLDN6) × CD3, in patients with late-stage solid tumors positive for the oncofetal antigen CLDN6 (NCT05262530) [134].

## Effective TCR signaling is beneficial for T cell activity

### Proximal signal transduction of TCR

**Fig. 4:**
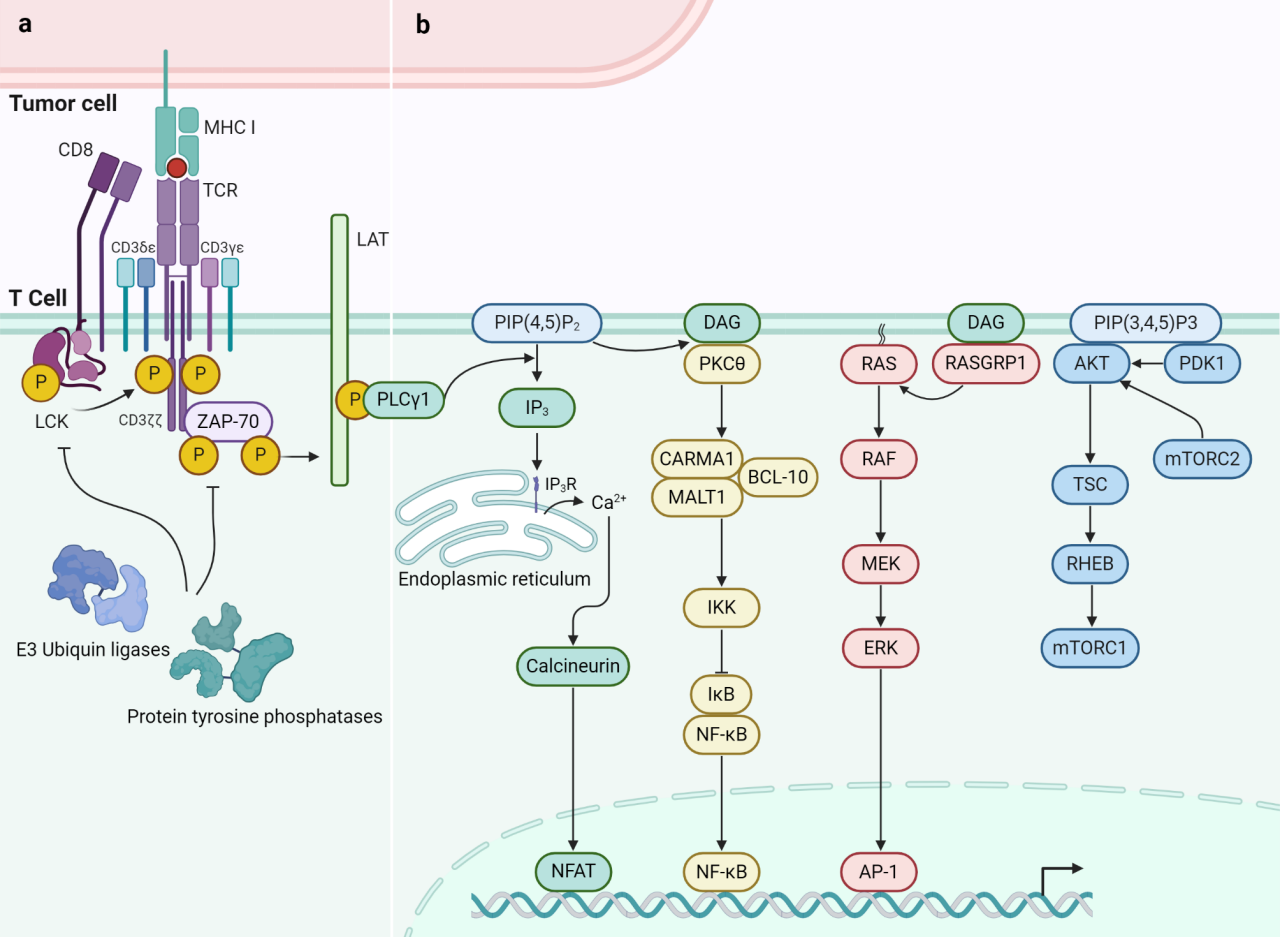
Signal transduction of TCR recognition of tumor antigens in CD8^+^ T cells. (**a**) The recognition of ligands by TCRαβ dimers triggers the transmission of information through the noncovalently associated CD3δε, CD3γε, and CD3ζζ modules, which contain cytoplasmic ITAMs phosphorylated by the Src-family kinase LCK. This phosphorylation event recruits ZAP-70, which is subsequently phosphorylated and activated by LCK. Activated ZAP-70 then phosphorylates the adaptor protein LAT, leading to the recruitment of additional signaling effectors. (**b**) TCR signaling induces extensive cellular changes in T cells, including the activation of transcriptional regulators, alterations in protein synthesis, and metabolic shifts. These changes guide the proliferation and effector differentiation of naive T cells. The key signaling pathways, as shown from left to right in the figure, are: [1]. PLCγ1 Pathway: Phosphorylation of LAT recruits PLCγ1, which hydrolyzes PIP2 to generate IP3 and DAG. IP3 induces calcium release from the endoplasmic reticulum, while DAG activates PKC, leading to the activation of transcription factors such as NFAT [2]. CARMA1/BCL10/MALT1 Pathway: The CARMA1/BCL10/MALT1 complex is activated downstream of LAT, leading to the activation of the NF-kB pathway. This pathway is crucial for the transcription of genes involved in T cell survival and proliferation [3]. RasGRP1/Ras/Raf/MEK/ERK Pathway: LAT phosphorylation also recruits RasGRP1, which activates the Ras/Raf/MEK/ERK signaling cascade. This pathway culminates in the activation of the transcription factor AP-1, promoting gene expression changes necessary for T cell activation [4]. PI3K/AKT/mTOR Pathway: LAT recruits PI3K, which generates PIP3, leading to the activation of AKT. AKT activation promotes mTORC2 and subsequently mTORC1 activation, which regulates protein synthesis and cell metabolism, essential for T cell growth and differentiation

The activation of CD8^+^ T cells largely depend on the signal transduction following TCR recognition of tumor antigens. The signal transduction process of antigen recognition is accompanied by conformational changes in the TCR-CD3 complex [135–137]. Therefore, the structural and functional integrity of the TCR-CD3 complex, as well as the coupling of LCK proto-oncogene, Src family tyrosine kinase (LCK) with signaling modules, is crucial for maintaining TCR signal transduction and activating T cells (Fig. 4a). The interaction of conserved polar residues within the TCRαβ TMD is key to the assembly of the TCR-CD3 modules [138]. The initial signal of antigen recognition is transmitted through the TCRαβ TMD to all modules of the TCR-CD3 complex, accompanied by structural changes in the signaling modules [139]. Understanding the structural changes in TM module interactions induced by ligand binding helps elucidate the mechanisms of TCR signal transduction and identify potential targets for enhancing TCR signaling. The membrane-embedded CD3ζζ contains a highly specific TM helical dimer interface, which is a key molecule coupling the TCR to intracellular signals [140]. In this process, the precise positioning of arginine within the TCRα TMD is critical [141]. Early experiments indicated that TCR binding triggers spatial changes between CD3ζζ subunits [142]. Subsequent studies defined a dynamic structural motion model of TMD related to the mechanobiology of the TCR-CD3 complex. This model revealed that force transmission induced by ligand recognition leads to a critical tilt of the TCRα TM helix, disrupting the interaction between Arg251 and CD3ζζ [143], causing the CD3ζζ tail to shift from lipid rafts to the cytoplasm, then providing tyrosine phosphorylation sites for LCK and promoting downstream signal transduction [144, 145]. Activated LCK recruits and phosphorylates ζ-chain-associated protein kinase 70 (ZAP-70) by phosphorylating immunoreceptor tyrosine-based activation motifs (ITAMs) [146]. The activated pZAP-70 further phosphorylates the key adaptor protein linker for activation of T cells (LAT) and promotes activation of TCR downstream signaling [147].

The strength of TCR signaling is a key determinant of CD8^+^ T cell anti-tumor responses. The availability of various proteins involved in TCR signal transduction can regulate T cell triggering, and their functional states under regulatory factors also influence the fate decisions of T cells post-triggering [148]. The protein tyrosine phosphatase Src homology region 2 domain-containing phosphatase (SHP) are key participants in the dephosphorylation reactions of the TCR signaling pathway of protein tyrosine phosphatases non-receptor (PTPN). They inhibit TCR signal transduction by directly or indirectly inactivating Src family kinases [149, 150]. The instability of SHP and PTPN is an important mechanism for enhancing TCR signal cascade reactivity. Studies have found that a serine/threonine kinase belonging to the STE-20 kinase family, Thousand-and-one-amino acid kinase 3 (TAOK3), acts as a rheostat for TCR signal transduction by regulating SHP-1 abundance, thereby determining the activation threshold of T lymphocytes [151]. Additionally, highly efficient and selective competitive inhibitors of PTPN active sites can achieve the loss of PTPN dephosphorylation function, thereby increasing T cell activation and recruitment [152]. Similarly, the suppressor of T cell signaling-1 (STS-1) encoded by *UBASH3B* can negatively transmit TCR signals by dephosphorylating pZAP-70 [153], and this biological function has been shown to be inhibited by the 2-(1 H)-quinolinone derivative Rebamipide [154]. Tense TCR signaling upregulates the expression of Cbl proto-oncogene, E3 ubiquitin protein ligase (CBL) in initial T cells [155], which then ubiquitinates and negatively regulates the LCK-ZAP-70-LAT axis to inhibit the transmission of proximal phosphorylation signals [156]. Understanding the negative feedback loop that exists between CBL and TCR signaling, which functions to temporally calibrate TCR signaling, helps selectively block CBL to enhance TCR signaling [157]. Furthermore, various LCK-dependent methods, such as CD61 and CD103 co-localization with TCR [158], CD146 homodimer-induced LCK autophosphorylation [159], and disulfiram increasing LCK kinase activity [160], have been shown to enhance proximal TCR signal transduction.

### Distal signal transduction of TCR

Downstream signals transmitted by the TCR signal complexes, in conjunction with co-stimulatory signals including CD28 and environmental signals, synergistically regulate the functional activation of CD8^+^ T cells. Multiple adaptive immunity signaling pathways participate in TCR signal transduction (Fig. 4b). When antigen-MHC complexes bind to TCRs, protein tyrosine kinases (PTKs) are activated, leading to the activation of phospholipase C-γ1. Phospholipase C-γ1 hydrolyzes membrane phospholipid phosphatidylinositol 4,5-bisphosphate, generating Inositol triphosphate (IP3) and diacylglycerol (DAG) [161]. IP3 binds to receptors on the endoplasmic reticulum membrane, causing the release of Ca^2+^ in the endoplasmic reticulum and activating calcium channels to control intracellular Ca^2+^ concentration [162]. Ultimately, the influx of Ca^2+^ activates the nuclear factor of activated T cells (NFAT) pathway through Ca^2+^-related proteins, regulating T cell metabolism and inducing cytokine production [163]. When TCR/CD28 connect, Phosphoinositide-dependent kinase 1 (PDK1) effectively activates the protein kinase C (PKC) θ through the phosphatidylinositol 3-kinase pathway [164]. Activated PKC phosphorylates serine residues in the caspase recruitment domain-containing membrane-associated guanylate kinase protein 1 (CARMA1) [165]. Then, B cell lymphoma/leukemia 10 (BCL10), and mucosa-associated lymphoid tissue translocation protein 1 (MALT1) are recruited, forming an active CARMA1-BCL10-MALT1 signal complex [166]. This promotes the activation of the IĸB kinase (IKK) complex and the degradation of IĸB, thereby allowing the NF-κB to translocate to the nucleus, initiating T cell activation, promoting T cell proliferation and cytotoxicity [167]. After TCR upstream signals are transmitted to Ras, Ras is activated by guanosine triphosphate (GTP) exchange, then sequentially activates Raf kinase, Mek kinase/Ras-extracellular signal-related kinase (ERK) kinase 1/2 and ERK, promoting the transcription of c-Fos, basic leucine zipper activating transcription factor (ATF)-like transcription factor (BATF), leading to the formation of the activator protein (AP)-1 complex, promoting the T cell activation program [168]. At the same time, ERK1/2-mediated SMAD family member 4 (SMAD4) Ser367 site phosphorylation induces SMAD4 nuclear translocation, promoting the activation and effector function of CD8^+^ T cells [169]. The mammalian target of the rapamycin (mTOR) pathway can be activated by various signals, regulating T cell survival and metabolic differentiation [170]. The mTOR signal transduction includes two different complexes, the activation of mTOR complex 1 (mTORC1) leads to the phosphorylation of S6 kinase 1 and the translation of 4E-BP1, while the activation of mTORC2 leads to the phosphorylation of Akt kinase [171]. The tuberous sclerosis complex (TSC)1 and 2 are tumor suppressors, they form heterodimers to regulate downstream signals in T cells [172]. When TCR stimulation and PKC induce TSC2 Ser1365 phosphorylation, the GTPase activity of the TSC1/2 complex is inhibited, leading to the activation of mTORC1 [171]. Interestingly, the expression of a phosphorylation-silent mutant TSC2-S1365A (SA) can significantly increase mTORC1 activation and T cell effector function [173]. T cells also have the potential to trigger specific effector functions independent of antigen receptor signal transduction. Analysis of chromatin accessibility in T cell subsets found that transcription factors RUNX1, PU.1, and BCL11B cooperatively recruit the chromatin remodeling complex mammalians switch/sucrose non-fermentable (mSWI/SNF) to the T_eff_ gene locus to establish a balanced chromatin landscape, supporting T cell activation [174].

## Reversing the exhausted state of CD8+ T cells

Effective CD8^+^ T cell responses are a key component of adaptive immune therapy for cancer. However, persistent TCR stimulation in solid tumors and the tumorigenic changes in the immune and metabolic landscape of the tumor microenvironment collectively drive naive CD8^+^ T cells to transition from T_ex_ precursor population through T_ex_ progenitor and T_ex_ intermediate subset to ultimately differentiate into T_ex_ terminally cells [175]. This primarily involves decreased cytokine secretion, sustained high expression of inhibitory receptors, metabolic changes, and unique transcriptional and epigenetic changes, ultimately leading to impaired effector function and reduced proliferation capacity of exhausted T cells [176–178]. The deepening understanding of the transcriptional, epigenetic, and metabolic characteristics that drive and maintain different exhaustion states of CD8^+^ T cells provide insights for designing immunotherapies that can reduce adverse events and improve tumor control.

**Fig. 5:**
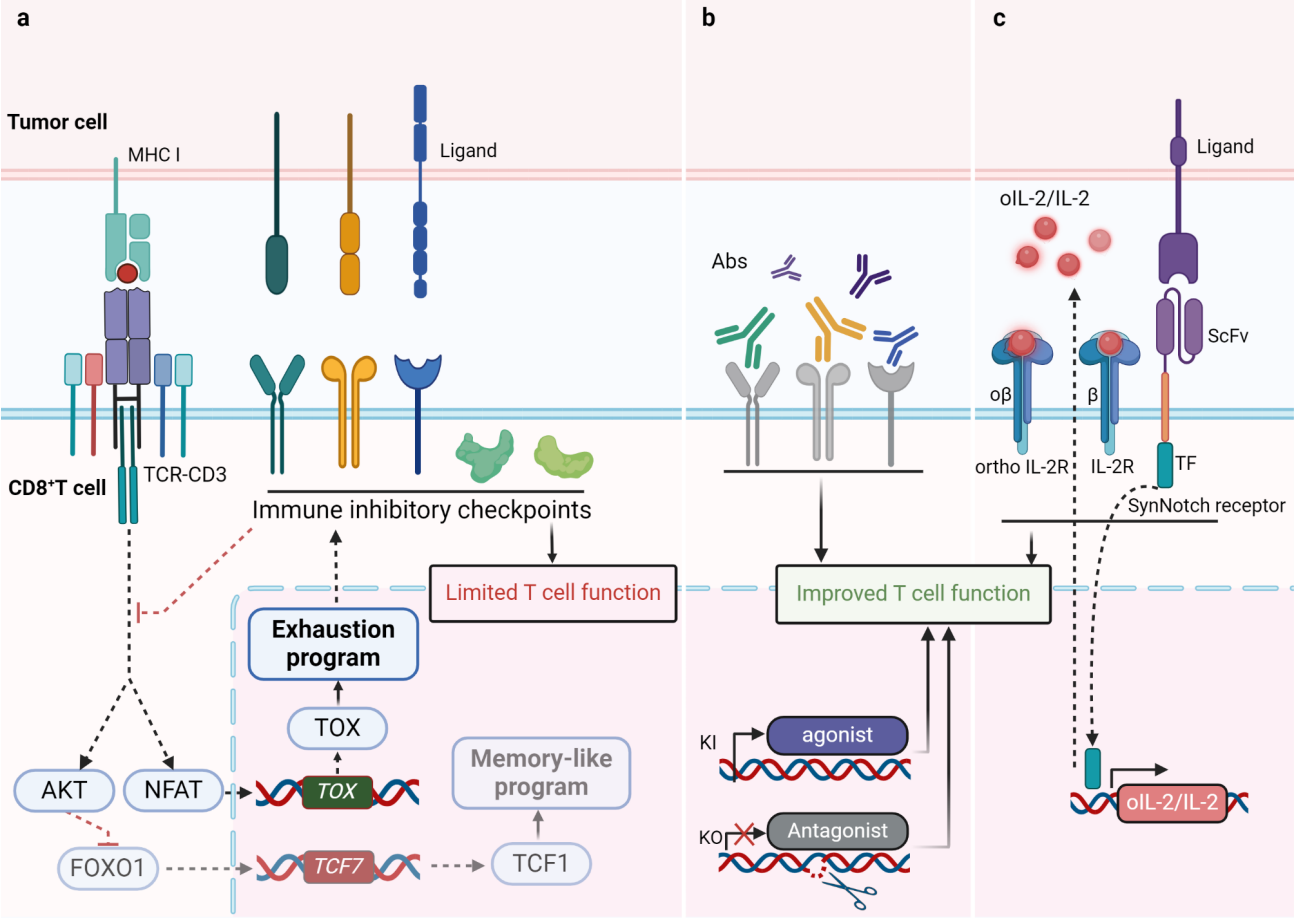
The occurrence and rescue of CD8^+^ T cell exhaustion program within solid tumors. **(a)** In solid tumors, chronic antigen stimulation signals from the TCR are transduced and transformed into increased accessibility of TOX and decreased availability of TCF1, ultimately driving the occurrence of the exhaustion program characterized by increased expression of immunosuppressive receptors, which limits the anti-tumor ability of CD8^+^ T cells. **(b)** By blocking inhibitory receptor signals, regulating the availability of intracellular immune checkpoints, and knocking out or knocking in of gene loci with weakened or enhanced function, the exhausted state of CD8^+^ T cells can be effectively reversed, restoring their anti-tumor function. **(c)** The use of orthogonal cytokine receptor engineering and synthetic Notch (synNotch) receptor engineering to increase the availability and specificity of cytokines for CD8^+^ T cells enable engineered CD8^+^ T cells to achieve effective expansion and improved adaptability

The process of naive CD8^+^ T cells becoming progressively exhausted after receiving antigen stimulation signals is accompanied by changes in the Transcription factor (TF) motif, mainly characterized by a decrease in the availability of T-cell factor 1 (TCF1) (encoded by the *TCF7* gene) and an increase in the availability of Thymocyte selection-associated high mobility group box protein (TOX) (encoded by the *TOX* gene) [179] (Fig. 5a). Compared with highly differentiated T cells, transferred T cell subsets originating from naive or stem cell-like cells exhibit stronger anti-tumor capabilities [180]. Among them, TCF1^+^ CD8^+^ T cells with stem cell-like characteristics are crucial for tumor control in cellular immunotherapy [4]. The HMG-box transcription factor TCF1, induced by the transcription factor Forkhead box O1 (FOXO1) binding to the *TCF7* site [181], regulates the memory-like program of CD8^+^ T cells and is key to the development and maintenance of stem cell-like memory precursor T cells and T_ex_ progenitor cells [182, 183]. The Inhibitor of DNA binding protein 2 (Id2) drives the stratified exhaustion of CD8^+^ T cells by disrupting the formation of the Tcf3-Tal1 transcription regulatory complex and recruiting the histone lysine demethylase 1 (LSD1), regulating the generation of T_ex_ progenitor cells and their transition to T_ex_ terminally cells, and promoting the maintenance of T cell stemness after adoptive cell therapy [184]. Another HMG-box transcription factor TOX is a central regulator of T cell exhaustion. When AP-1 is absent, the transcription factor NFAT imposes a negative feedback program on exhausted T cells, initiating *TOX* transcription and converting persistent stimulation signals into Tex-related transcriptional and epigenetic developmental programs [185, 186]. The interaction between BATF and interferon regulatory factor 4 (IRF4) can reduce the expression of TOX, promote the effector differentiation of the transcriptional and epigenetic landscape of exhausted T cells [187], thereby improving the anti-tumor response of engineered T cells [188].

### Blocking immune checkpoint

Co-inhibitory receptors on the surface of T cells are a routine immune checkpoint in cancer immunotherapy and are key to enabling cancer cells to evade immune recognition and attack. Immune checkpoint blockade (ICB) therapy, by blocking the IRs signals on the surface of exhausted T cells, enables them to restore and maintain effector function to clear tumors, showing promise in the treatment of an increasing number of solid tumors [189] (Fig. 5b). Programmed death protein-1 (PD-1) and cytotoxic T lymphocyte antigen 4 (CTLA4) are the most well-known immune checkpoints, promoting immune tolerance by binding to their ligands PD-L1 and B7 family molecules respectively, and therapeutic antibodies targeting them have greatly changed cancer immunotherapy in the past [190]. Recent research has revealed a new structure-function relationship of PD-1, in which PD-1 and its ligand drive PD-1/PDL-1 cis-dimerization through non-covalent binding of the transmembrane structural domain, and enhance the functional inhibition of CD8^+^ T_eff_ [191]. Therefore, manipulating the oligomeric state of the PD-1 axis is expected to provide an additional way to enhance cancer immunotherapy. Nevertheless, a portion of cancer patients still show low responsiveness to treatments targeting PD-1 and CTLA4, which has also promoted the exploration of the next generation of inhibitory receptors. Lymphocyte activation gene-3 (LAG3) is a co-inhibitory receptor highly expressed on exhausted T cells [192]. It colocalizes with the TCR during early T cell activation events [193], and increases the acidity of the immunological synapse through its cytoplasmic tail’s conserved EP motif, which is a tandem repeat of glutamic acid-proline sequences [194]. This leads to the uncoupling of LCK from CD8 and TCR-CD3 complexes, ultimately suppressing T cell activation [195]. T cell immunoglobulin-3 (Tim-3) is selectively expressed on CD8^+^ cytotoxic T lymphocytes (CTL) and is upregulated during CD8^+^ CTL dysfunction or exhaustion [196]. By interacting with its heterophilic ligand Carcinoembryonic antigen-related cell adhesion molecule-1 (CEACAM1), Tim-3 prevents the phosphorylation of TCR signaling modules within effector T cells, ultimately inhibiting T cell activation [197]. T cell immunoglobulin and immunoreceptor tyrosine-based inhibitory motif (ITIM) domain (TIGIT) is expressed on activated T cells, mainly connects to ligand CD155 in a CD226-independent manner, and forms nanoclusters located at the TCR immune synapse, inhibiting the acquisition of effector function and proliferation of effector T cells by downregulating the expression of TCR complex-related proteins and the central regulatory factors of the TCR signal cascade [198]. B and T lymphocyte attenuator (BTLA), a lymphocyte-specific cell surface co-inhibitory receptor, inhibits antigen-driven T cell activation and proliferation by binding to herpesvirus entry mediator (HVEM), and subsequently recruits SHP1/2 through the typical cytoplasmic domain of ITIM and immunoreceptor tyrosine-based activation motif (ITSM) [199, 200]. The clinical control of tumors has been enhanced to varying degrees in solid tumor therapy by blocking LAG3 [201, 202], Tim-3 [203, 204], TIGIT [205, 206], BTLA [207] to improve the adaptability of CD8^+^ tumor infiltrating lymphocytes (TILs). The CD200-CD200R axis is a potential target for antitumor immunomodulation. Typically, CD200^+^ CD8^+^ T_eff_ exhibit improved antitumor functionality following immune checkpoint blockade therapy [208]. However, upon chronic antigen stimulation, the inhibitory receptor CD200R1 is upregulated on the surface of CD8^+^ T_eff_ and suppresses T cell activity through its interaction with the ligand CD200 [209]. Selective blockade of CD200R1 can help restore the antitumor function of CD200R1^+^ CD8^+^ T_eff_ [210]. V domain immunoglobulin suppressor of T cell activation (VISTA), a B7 family immune checkpoint protein expressed on T cells, drives T cell inhibition by binding to homologous ligands [211], it has been proven in tumor models that VISTA blockade can enhance the proliferation and cytotoxic function of CD8^+^ T_eff_ [212]. Recently, a new immune checkpoint I-type single-pass membrane protein (ITPRIPL1) was characterized in tumors. The ITPRIPL1-CD3ε axis formed by the binding of ITPRIPL1 as a ligand on the surface of tumor cells with CD3ε can inhibit TCR-CD3 complexes signal transmission. Therapeutic antibodies targeting ITPRIPL1 can effectively enhance the cytotoxicity of tumor-specific T cells and achieve tumor growth control and are expected to be promising therapeutic targets for various types of tumors [213]. The development of bispecific antibody drugs that simultaneously target inhibitory receptors/ligands and another inhibitory receptor or co-stimulatory receptor, by selectively targeting immune checkpoints to rescue T cell exhaustion, provides additional options for enhancing the anti-tumor ability of CD8^+^ T cells [214]. More importantly, engineered synthetic ‘switch’ receptors-T cells that fuse inhibitory receptor structural domains to activation structural domains, including TIGIT-CD28 [215], CD200R-CD28 [216], TIM-3-CD28 [217], PD-1-CD28 [218], have shown superior anti-tumor capabilities in preclinical models or clinical trials, providing a realistic basis for remodeling solid tumor ACT.

In addition to cell surface inhibitory receptors, intracellular checkpoints are gradually being discovered as emerging targets (Table 1). Odd-skipped related 2 (Osr2) is a mechanoresponsive transcription factor, which is selectively induced in terminally exhausted tumor-specific CD8^+^ T cell subsets by TCR signal transduction and biomechanical stress mediated by the Piezo1/Ca^2+^/cyclic adenosine monophosphate(cAMP)-response element binding protein (CREB) axis. Subsequently, Osr2 recruits histone deacetylase 3 (HDAC3) to downregulate Histone H3 Lysine 27 acetylation (H3K27ac) signaling, inhibiting the expression of cytotoxic genes and exacerbating T cell exhaustion [219]. In solid tumor models, the absence of Osr2 can alleviate the exhausted state of transferred tumor-specific T cells and enhance their control of tumors, suggesting that Osr2, as a biomechanical checkpoint, holds promise as a new target for solid tumor immunotherapy. The co-stimulatory receptor CD226 is a key component of CD8^+^ T cell activation within tumors, and it can inhibit targets expressing CD112 and CD155 [220]. Overexpression of the transcription factor Eomesodermin leads to the loss of CD226 in CD8^+^ TILs, thereby blocking the effector program of T cells, and can serve as an intracellular immune checkpoint [221]. Regulating the expression and signal transduction of membrane receptors in CD8^+^ T cells through the control of post-translational processes of membrane proteins is an effective method to alter the anti-tumor effects of CD8^+^ T cells. The extracellular domain protease A disintegrin and metalloprotease 17 (ADAM17) inhibits the perception of interleukin-2 (IL-2) signals by CD8^+^ T cells by cleaving the membrane IL-2 Receptor β (CD122), thereby reducing the proliferation and effector differentiation of CD8^+^ T cells. In solid tumor models, the knockout or inhibition of ADAM17 can significantly upregulate the expression of CD122 on the membrane of CD8^+^ T cells and enhance their anti-tumor capabilities [222]. Additionally, CD8^+^ T cells have also generated their own regulatory signal transduction axis. In tumor-infiltrating exhausted CD8^+^ T cells, the immunosuppressive factor fibrinogen-like protein 2 (Fgl2) is expressed and positively regulated by FcγRIIB, which is the only inhibitory IgG-Fc receptor expressed in highly differentiated effector CD8^+^ T cells. Meanwhile, Fgl2 supports the suppression of the anti-tumor reactivity of CD8^+^ T cells by inducing caspase 3/7-mediated apoptosis through interaction with FcγRIIB [223]. It has been confirmed in mouse and human tumor models that inhibitory regulation of the Fgl2/FcγRIIB axis can effectively rescue disabled antigen-specific CD8^+^ T cells.

**Table 1:** Immune checkpoints associated with enhanced anti-solid tumor efficacy of CD8^+^ T cells

Checkpoints	Interaction site	Effect on T cells	Treatment method	Research progress	Reference
PD-1	PD-L1/PD-L2	Negatively regulating TCR signaling	Drugs: Nivolumab/Pembrolizumab	Approved	[190]
CTLA4	B7 family molecules	Negatively regulating TCR signaling	Drugs: Ipilimumab	Approved	[343]
LAG3	Galectin-3/FGL1	Negatively regulating TCR signaling	Drugs: Relatlimab	Approved	[344]
TIM3	CEACAM-1	Negatively regulating TCR signaling	Antibody	Clinical trials (NCT03489343, NCT04145622)	[203]
TIGIT	CD155/CD112	Negatively regulating TCR signaling	Antibody	Clinical trials (NCT04145622, NCT04540211, NCT05394168)	[205]
BTLA	HVEM	Negatively regulating TCR signaling	Antibody	Clinical trials (NCT04278859, NCT05427396, NCT04137900)	[207]
VISTA	VSIG3	Negatively regulating TCR signaling	Antibody	Clinical trials (NCT05082610, NCT04137900)	[212]
CD200R1	CD200	Inhibiting cytokine production	Blocker	Preclinical study	[210]
LRIG1	VISTA	Negatively regulating TCR signaling	Blocker	Preclinical study	[347]
PSGL-1	P-selectin/VISTA	Cooperating with VISTA signaling	Blocker	Preclinical study	[226]
CD3ε	ITPRIPL1	Negatively regulating TCR signaling	Blocker	Preclinical study	[213]
Fgl2	FcγRIIB	Inducing apoptosis	Down-regulated expression	Preclinical study	[223]
ADAM17	IL-2Rβ	Inhibiting IL-2 signaling	Down-regulated expression	Preclinical study	[222]
THEMIS	BTLA	Inhibiting BTLA signaling	Up-regulated expression	Preclinical study	[224]
Osr2	HDAC3	Inhibiting cytokine production	Reduced mechanical stress; Down-regulated expression	Preclinical study	[219]
Eomes	Regulatory elements of CD226	Downregulating CD226 expression	Down-regulated expression	Preclinical study	[221]

The operability of immune checkpoints is mainly described as a simple switching mechanism dependent on homologous ligand interactions, depending on intracellular factors that regulate the signal threshold of these receptors to inhibit T cell responses and is constrained by intracellular signal attenuators. T-lineage protein thymocyte-expressed molecule involved in selection (THEMIS) is an intracellular signal attenuator in T cells. It is recruited to the cytoplasmic structural domain of BTLA, and by interacting with growth factor receptor-bound protein 2 (GRB2) and SHP-1, it stabilizes the oxidation of SHP-1, thereby inhibiting BTLA signal transmission, ultimately promoting and maintaining the development of CD8^+^ T cells [224]. P-selectin glycoprotein-1 (PSGL-1) is an intrinsic immune checkpoint regulatory molecule of T cell exhaustion. It connects with TCR and inhibits ZAP-70 phosphorylation to block TCR proximal signal transmission by maintaining the expression of Ubiquitin-associated and SH3 domain-containing protein B, thereby limiting the activation of CD8^+^ T cell effector function and inducing the transition of T cells to exhaustion [225]. In addition, PSGL-1 can selectively bind to VISTA in the acidic tumor microenvironment, mediating the suppression of T cell activity [226]. Deletion or blockade of PSGL-1 allows CD8^+^ T cells to gain enhanced TCR sensitivity and metabolic characteristics, promoting their effector function and stem cell differentiation, and achieving successful tumor control in solid tumors resistant to PD-1 blockade [225].

### Gene editing enhances the intrinsic adaptability of CD8+ T cells

Enhancing the adaptability of engineered therapeutic T cells under chronic antigen stimulation is also a promising strategy to improve anti-tumor efficacy. Due to the ability of the CRISPR/Cas9 gene editing system to precisely inactivate or knock-in target genes at the genomic DNA level, functional genomic screening based on CRISPR/Cas9 has been used to identify T cell therapy targets [181, 227] (Fig. 5b). Suppressor of cytokine signaling 1 (SOCS1), by negatively regulating the Janus kinase (JAK)/signal transducer and activator of transcription (STAT) signaling pathway, inhibits the accumulation of central memory T cells and T_eff_ subsets in cancer and has been identified as an optimal T cell enhancement target. Targeting the SOCS1 SH2 structural domain with single guide RNA (sgRNA) via CRISPR/Cas9 to inactivate *Socs1* in engineered T cell can enhance the reactivity of transferred T cells to cytokines and inhibit tumor growth in mice [228]. Intrinsic inflammatory regulatory factors Regnase-1, encoded by zinc finger CCCH-type containing 12A (*ZC3H12A*) and Roquin-1, encoded by ring finger and CCCH-type domains 1 (*RC3H1*) in T cells limit the expression of inflammatory genes by targeting and degrading the 3’-untranslated regions (UTRs) of inflammatory gene transcripts [229]. The absence of Regnase-1 reprograms CD8^+^ T cells to increase their inflammatory activity and persistence, thereby enhancing control of solid tumors [230], and co-knockout with Roquin-1 can significantly improve this anti-tumor function [231]. Protein tyrosine phosphatase PTPN2, encoded by *PTPN2*, regulates the generation of terminal exhaustion T cell subsets by weakening type I interferon signals. The absence of PTPN2 can restore the cytotoxicity and proliferative ability of exhausted T cells in a cytokine-independent manner [232], and enhance their control of solid tumors [150]. Moreover, small molecule inhibitors of the PTPN active site have achieved similar effects in preclinical models [233]. RAS P21 protein activator 2 (RASA2), encoded by *RASA2*, is a RAS GTPase-activating protein, which gradually increases in expression in CD8^+^ T cells under chronic antigen exposure. In the treatment of solid tumors with T cells in mice, the ablation of *Rasa2* enhances the response of TIL to TCR stimulation through the mitogen-activated protein kinase (MAPK) signaling pathway, increases TIL antigen sensitivity, promotes its immune reprogramming, and ultimately achieves a more persistent effector function [234]. The family with sequence similarity 49 member B (FAM49B), encoded by CYFIP related Rac1 interactor B (*CYRIB*), is a regulator of proximal TCR signaling that inhibits the activation of CD8^+^ T cells by suppressing the active form of the small GTPase Rac and cytoskeletal actin dynamics [235]. The genetic elimination of *CYRIB* or the interaction between FAM49B and Rac can block the negative regulatory effect of FAM49B on T cells. The p38 kinase, encoded by *MAPK14*, has been identified as a central negative regulator of the effective anti-tumor phenotype characteristics of CD8^+^ T cells. Inhibiting *Mapk14* is an effective method to promote the functional phenotype differentiation of T cells and enhance the anti-tumor effect of engineered T cells in mice [236]. The accessibility of the inositol requiring mutant 80 (INO80) and BRG1 or BRM-associated factor (BAF) chromatin remodeling complexes is related to the persistence of CD8^+^ T cells in tumors. The genetic deletion of chromatin remodeling factors, especially the BAF complex member *Arid1a*, can reduce the epigenetic characteristics related to CD8^+^ T cell exhaustion in mouses bearing melanoma, thereby increasing their function and persistence [237]. In addition to the majority of negative regulatory genes, some gain-of-function (GOF) gene targets within CD8^+^ T cells have also been screened. Proline dehydrogenase 2 (PRODH2), encoded by *PRODH2*, has been identified as a key regulatory enzyme for reprogramming proline metabolism in primary CD8^+^ T cells. Moreover, targeted knock-in of *Prodh2* can reshape the metabolic group, transcriptome, and immune function of engineered T cells, thereby enhancing the anti-tumor efficacy in mice [238]. The caspase recruitment domain-containing protein 11 (*CARD11*)-phosphoinositide-3-kinase regulatory subunit 3 (*PIK3R3*) fusion gene, found in human skin T lymphocyte cancer, regulates T cell proliferation and differentiation by enhancing CARD11-BCL10-MALT1 complex signaling [239]. CD8^+^ engineered T cells carrying the *Card11*-*Pik3r3* fusion gene have been shown to have increased functionality and stemness in animal model. Subsequently, in a screening of the whole genome of primary human T cells for functional key regulatory targets using sgRNA lentiviral infection with Cas9 protein electroporation (SLICE), casitas B-lineage lymphoma proto-oncogene B (*CBLB*) and *CD5* were identified as negative regulators, and lymphocyte cytosolic protein (*LCP*) was identified as a positive regulator in T cell activation [240] (Table 2).

**Table 2:** Gene-editable targets for enhancing anti-solid tumor activity within CD8^+^ T cells

Targets	Physiological effect in T cell	Gene editing	Tumor	Efficacy	Clinic trial	Reference
SOCS1	Inhibiting cytokine reactivity	KO	Melanoma in mice	Suppressing tumor growth	Advanced solid tumors (NCT06237881)	[228]
Regnase-1/Roquin-1	Inhibiting inflammatory phenotype	KO	Melanoma in mice	Improving survival rate	N/A	[231]
PTPN2	Inducing differentiation of exhausted phenotype	KO	Melanoma in mice	Suppressing tumor growth	N/A	[150]
RASA2	Inhibiting TCR antigen sensitivity	KO	Melanoma in mice	Improving survival rate	N/A	[234]
P38	Inhibiting the differentiation of effector phenotype	KO	Melanoma in mice	Improving survival rate	N/A	[236]
ARID1A	Inducing the occurrence of terminal exhaustion phenotype	KO	Melanoma in mice	Suppressing tumor growth	N/A	[237]
CBLB	Inhibiting proximal TCR signaling	KO	Colon carcinoma in mice	Suppressing tumor growth	N/A	[345]
CD5	Inhibiting proximal TCR signaling	KO	Pancreatic carcinoma in mice	Suppressing tumor growth	N/A	[346]
PRODH2	Inducing immune metabolic remodeling	KI or OE	Breast carcinoma in mice	Suppressing tumor growth	N/A	[238]
CARD11-PIK3R3	Inducing effector and memory phenotype differentiation	KI or OE	Melanoma in mice	Improving survival rate	N/A	[239]

KO: Knock-out, KI: Knock-in, OE: Overexpression, N/A: Not Applicable

Recently, a programmable RNA targeting platform, multiplexed effector guide arrays (MEGA), was developed. This platform uses RNA-guided CRISPR-Cas13d with RNA targeting activity to perform highly multiplexed, quantitative, and reversible perturbation of the transcriptome of primary human T cells [241]. Combined with CRISPR combination screening, it can achieve synchronous regulation of paired regulatory factors affecting T cell function. Another modular platform for constructing DNA knock-in libraries, modular pooled knock-in screening (ModPoKI), evaluates hundreds to thousands of different T cell structures by merging hundreds of TFs and synthetic surface receptors (SRs) into different TCR-T cells, obtaining synthetic sequences targeting interested genomic binding sites [242]. In engineered T cell immunotherapy, this large-scale ModPoKI screening is a powerful method to accelerate cell state programming, helping to discover complex gene structures that program cell function, and successfully revealing the role of overexpressed TFAP4 and BATF in promoting the function and improving adaptability of engineered T cells in mouses bearing leukemic. In addition to genome editing, CRISPR-based base editing technology can be utilized for analyzing single-nucleotide variants (SNVs) in engineered T cells. Through this approach, gain-of-function mutations in genes encoding phosphatidylinositol-4,5-bisphosphate 3-kinase catalytic subunit delta (PIK3CD) and its related regulatory subunits have been identified to significantly enhance the anti-tumor capability of engineered CD8^+^ T cells ex vivo, which could potentially aid in guiding the engineering design of cell-based immunotherapy [243]. Engineered T cells expressing CD47 (Q31P) mediate an increase in macrophage-T cell crosstalk while evading macrophage phagocytosis, ultimately achieving more durable anti-tumor capabilities [244], it is anticipated to be rapidly constructed using CRISPR-based base editing technology.

### Cytokine engineering reduces the occurrence of CD8+ T cell exhaustion

The binding of TCR/auxiliary receptors induces the activation of biochemical signaling pathways. These signaling pathways, in conjunction with signals from cytokine receptors, guide the outcome of the response. The lack of success of engineered TCR-T in solid tumor treatment may be related to the lack of available cytokine signals. Selectively enhancing the targeting of cytokines may provide new ideas for enhancing strategies for improved TCR-T cells [245] (Fig. 5c). However, some adoptive cell therapies supplemented with cytokines show off-target or systemic toxicity [246]. Therefore, strategies using cytokine engineering to actively regulate ILs/IL receptors (ILRs) expression are used to design the next generation of therapeutic T cells. An engineered IL-2 agonist, named H9T, can effectively bind to IL-2Rβ even in the absence of IL-2Rα [247]. It drives T cell proliferation while maintaining the expression of TCF-1, thereby promoting the maintenance of T cell stemness and preventing terminal differentiation of T cells [248]. Ultimately, it significantly prolongs the survival time of melanoma mouse models. Using orthogonal gene engineering, allowing T cells to express ortho IL-2Rβ, so that they can be selectively targeted by ortho IL-2 [249], or allowing T cells to secrete an IL-2 variant that can bind to IL-2Rβγ and the alarmin IL-33, can make adoptively transferred engineered T cells far from exhaustion and maintain superior effector function, ultimately improving the survival rate in mouses bearing advanced colon tumor [250]. Synthetic Notch (synNotch) receptor is a transmembrane receptor that converts signals from tumor-derived ligand-recognized extracellular structures into the release of designated transcription regulatory factors in intracellular structural domains, with orthogonality and flexibility [251]. Tumor-specific synNotch receptor engineered T cells based on the synNotch pathway can independently of T cell activation, rely on bypass signaling pathways to achieve IL-2 secretion, thereby enhancing T cell proliferation and effects while reducing potential cell toxicity risks [252]. It was demonstrated in immunosuppressive pancreatic cancer mouse models with significantly prolonged survival. In addition, the upregulation of membrane-tethered IL-15/IL-21 co-expression in engineered T cells [253], the selective expression of homodimeric IL-21R replaced by CD34 extracellular structural domain [254], and the fusion protein of IL-2 with IL-2Rα with selective affinity [255], can all effectively reduce the exhaustion of therapeutic T cells and enhance their anti-tumor function in different preclinical murine solid tumor models.

## Metabolic programming regulation of CD8+ T cell functionality

**Fig. 6:**
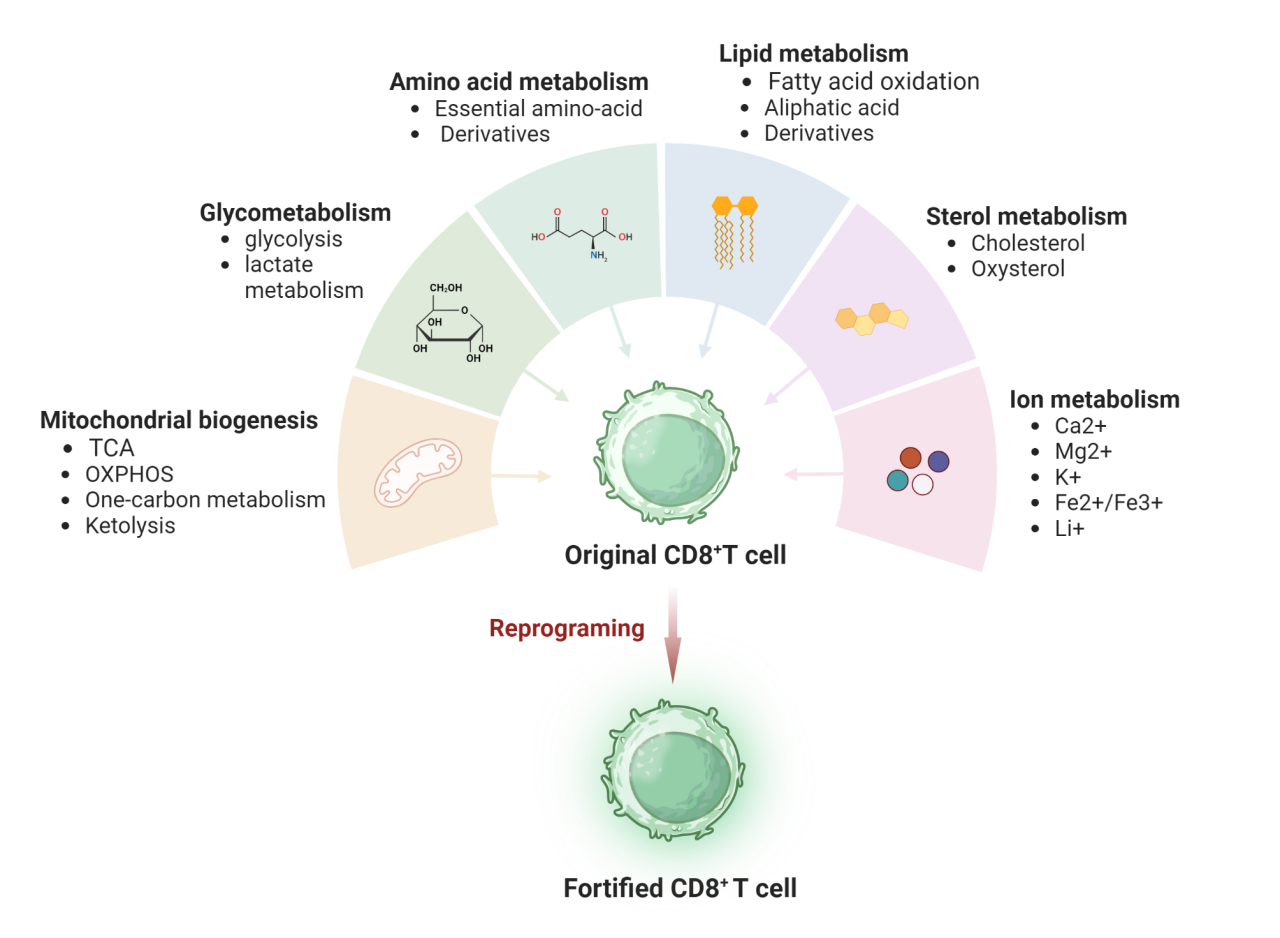
Metabolic pathways enhancing the anti-tumor function of CD8^+^ T cells. Appropriate metabolic program modulation of quiescent or dysfunctional CD8^+^ T cells can promote T cells to exit quiescence or rejuvenate exhausted T cells. The reprogramming pathways mainly include mitochondrial biogenesis, glucose metabolism, amino acid metabolism, lipid and sterol metabolism related to nutrient synthesis, and ion metabolism related to the regulation of cellular life activities

Antigen recognition activates the TCR-CD3 complex signaling pathway, driving intricate metabolic programs within T cells. The balance of energy and material metabolism within T cells largely determines the activation and maintenance of their antitumor activities. Therefore, understanding the processes of energy production and biosynthesis in T cells, as well as the impact of these processes on T cell differentiation and functional maturation, will be beneficial in identifying new effective targets to enhance antitumor immunotherapy. Subsequently, by reprogramming T cell metabolism to regulate these proven “targets”, the transcriptome of T cells can be shifted towards characteristics that favor antitumor immunity. This represents an effective strategy for modifying T cells. (Fig. 6).

### Metabolism of mitochondria

The tricarboxylic acid cycle (TCA) that occurs within mitochondria serves as the hub of cellular energy metabolism and is the final pathway for the metabolism of sugars, fats, and amino acids [256]. In CD8^+^ T cells, the TCA cycle, driven by glucose and glutamine, serves as the principal metabolic pathway for sustaining mitochondrial energy metabolism and intratumoral migration [257]. Variations in the expression of key metabolic enzymes and the availability of intermediate metabolites throughout this process can influence the homeostasis of energy metabolism [258, 259]. Reduced equivalents (FADH/NADH/NADPH) derived from glycolysis, the TCA cycle, and the pentose phosphate pathway participate in oxidative phosphorylation in mitochondria to directly supply energy for almost all life activities of T cells [260]. The survival and proliferation of CD8^+^ T cells depend on oxidative phosphorylation [261–263], and controlling the occurrence and progression of reduced equivalent generation reactions to regulate oxidative phosphorylation is an effective strategy. Among them, mTORC1 is an important regulatory factor, which induces the expression of transcription factors, such as IRF4/MYC Proto-Oncogene (MYC), and changes the metabolic state of T cells through transcription, translation, and post-translational modification [264–266]. Another key metabolic checkpoint is the mitochondrial pyruvate carrier (MPC), which transports pyruvate in the cytoplasm to mitochondria to enhance oxidative phosphorylation [267]. Inhibition of MPC will inhibit aerobic oxidation in T cells so that CD8^+^ T cells cannot maintain complex life activities [268]. At the same time, T cell energy metabolism is also closely related to TCR signals and CD28 co-stimulatory signals [269–272]. Mechanistically, TCR signals and CD28 co-stimulatory signals mediate the fusion of mitochondrial inner and outer membranes, driving more efficient electron transport chains to enhance oxidative phosphorylation [273, 274].

Mitochondria are also important sites for material metabolism. When T cells are activated, the mitochondrial one-carbon metabolism pathway is enhanced [275]. Serine is the main amino acid involved in one-carbon metabolism [276], and is crucial in promoting T cell proliferation and activation [275, 277]. The smooth entry of serine into mitochondria is the key to this metabolic process, and Sideroflexin1 (SFXN1) has been proven to be a key protein transporting serine into mitochondria [278]. The methionine cycle provides methyl for various biochemical reactions. S-adenosylmethionine (SAM) and its downstream metabolite 5-methylthioadenosine (MTA) can induce the occurrence of CD8^+^ T cell exhaustion program. And inhibition of MAT2A, a key enzyme for SAM synthesis, can effectively improve CD8^+^ T cell function in mouses bearing hepatocellular carcinoma and increase their overall survival [279]. Ketone bodies (KBs) are produced by the β-oxidation of fatty acids in mitochondria and are essential fuels for maintaining CD8^+^ T cell effector function. Endogenous KBs oxidation (ketolysis) drives CD8^+^ T cell effector-like metabolism. Mechanistically, KBs are preferentially utilized to supply substrates for the TCA cycle, thereby directly enhancing the TCA-dependent metabolic pathways of CD8^+^ T_eff_ cells [280].

Mitochondrial biogenesis maintains the energy and metabolic homeostasis within CD8^+^ T cells, and proliferator-activated receptor (PPAR) gamma coactivator 1α (PGC-1α)—a transcriptional activator that promotes mitochondrial and nuclear interaction [281]—is a key regulatory factor [282]. The inhibitory tumor microenvironment often causes the expression of PGC-1α in TILs to be suppressed, leading to T cell disability [283]. The application of overexpressing PGC-1α in CD8^+^ T cells through genetic engineering can effectively enhance its anti-tumor effect in mouses bearing lung carcinoma [284, 285]. In addition, 4-1BB co-stimulation can upregulate PGC1α expression, and activate ATF2 through the p38-MAPK pathway, thereby enhancing mitochondrial fusion and biogenesis, and ultimately promoting the immunity and memory of CD8^+^ T cells in mouses bearing melanoma [286].

### Metabolism of glucose and amino acid

Glucose and amino acid metabolism are crucial for T cell activation [261, 287]. Activated T cells regulate glucose uptake from the TME through Glucose transporter 1 (GLUT1) [288]. Then, glucose undergoes glycolysis to generate pyruvate, which can be transported to mitochondria to participate in the TCA [287]. Adjusting the availability of metabolic products and key metabolic enzymes of these biochemical reactions can guide T cell metabolic reprogramming. Phosphoenolpyruvate maintains T cell receptor-mediated Ca^2+^-NFAT signal transduction and effector function by inhibiting sarco/endoplasmic reticulum Ca^2+^-ATPase (SERCA) activity [289]. Phosphoenolpyruvate carboxykinase 1 (PCK1) not only enhances the impact of phosphoenolpyruvate on T cell function but also regulates T cell differentiation [290]. Pyruvate, the end product of glycolysis, is oxidized to lactate by lactate-dehydrogenase (LDH), reducing TCA substrate supply while increasing acid load. Moreover, lactate is a double-edged sword. On the one hand, physiological concentrations of lactate inhibit histone deacetylase activity, and increase H3K27 acetylation, thereby upregulating *Tcf7* expression, ultimately increasing CD8^+^ T cell stemness and delaying colon tumor growth in mouses [291]. On the other hand, the Warburg effect of tumor cells that cells prefer to metabolize glucose to lactate even in the presence of oxygen, leads to a high lactate load in the TME [292]. TIL interaction with lactate in the TME is mediated by monocarboxylate transporter 1 (MCT1), a bidirectional proton-assisted transport protein encoded by *SLC16A1*, the directionality of which is determined by the concentration of substrate and H^+^ [293]. High concentrations of lactate promote the reduction of NAD^+^ to NADH, causing changes in NAD^+^ dependent enzyme-catalyzed reactions [294], thereby reducing the production of metabolites needed for T cell proliferation, ultimately limiting CD8^+^ T cell effector function [295, 296]. It has been substantiated that the stem cell-like memory differentiation of adoptively transferred CD8^+^ T cells can be effectively augmented by the inhibition of glycolysis [297, 298], an effect that is intimately associated with the preservation of FOXO1 activity within these T cells.

Glutamine serves not only as the primary fuel for early CD8^+^ effector T cells but also as a crucial biosynthetic substrate. Glutamic-oxaloacetic transaminase 1 (Got1), which regulates *de novo* synthesis of aspartate from glutamine, is essential for sustaining the expansion and effector function of CD8^+^ effector T cells [299]. One of the essential amino acids, leucine, activates mTORC1 in a large amino acid transporter 1 (LAT1)/CD98-dependent manner to induce metabolic reprogramming, promoting CD8^+^ T cell activation [300]. Non-essential amino acids are usually synthesized from glucose or glutamine metabolic derivatives, participating in T cell metabolic activities [301]. The metabolic activity of amino acids is accompanied by the accumulation of metabolites. Kynurenine, a tryptophan metabolite, can be taken up by T cells through LAT1/CD98 regulation [302] and has been shown to inhibit T cell proliferation [303]. The enzyme co-factor tetrahydrobiopterin (BH4) can rescue the inhibition of kynurenine on T cells, upregulating the expression of GTP cyclohydrolase 1 (GCH1) and sepiapterin reductase-key enzymes in the BH4 synthesis process-significantly enhancing CD8^+^ T cell anti-tumor activity and controlling tumor growth in mouse bearing breast tumor [304]. L-arginine, a derivative of intracellular arginine metabolism, can enhance T cell adaptability, control tumor growth, and prolong overall survival in mouses bearing melanoma [305]. Mechanistically, L-arginine induce a shift in T cell respiration from glycolysis to oxidative phosphorylation. The key transcriptional regulators mediating this process are Bromodomain adjacent to zinc finger domain 1B (BAZ1B) and PC4 and SFRS1 interacting protein 1 (PSIP1). In CD8^+^ T cells, the absence of taurine, a cysteine metabolic derivative, increases endoplasmic reticulum stress and induces ATF4 transcription. This, in turn, activates immune checkpoint gene expression, leading to CD8^+^ T cell exhaustion [306].

### Lipid and sterol metabolism

Glucose-derived pyruvate is synthesized into acetyl-CoA in mitochondria through the TCA cycle [307], and acetyl-CoA enters the mevalonate pathway to promote the synthesis of lipids and sterols [308]. When T cells encounter nutrient stress, AMP-activated protein kinase (AMPK), a serine/threonine kinase, is activated [262] then induces the biosynthesis of lipids and sterols in a mTORC1-dependent manner by upregulating sterol regulatory element-binding transcription factor (SREBP)1/2, maintaining the energy material supply for CD8^+^ T cells [309]. SREBP cleavage-activating protein (SCAP) is an activator of SREBP, which can promote the proliferation and functional activation of CD8^+^ T cells [310].

In solid tumors, rapidly proliferating tumor cells disproportionately compete with TILs for energy metabolites such as glucose and amino acids, leading to impaired T cell metabolic status and inability to maintain the energy synthesis required for survival and anti-tumor activity [17, 311]. At this time, fatty acid oxidation (FAO) becomes an effective way to produce energy, and it has been proven in mouses bearing melanoma that TILs rich in FAO are more effective in controlling cancer [312]. Lipids not only participate in energy synthesis but also are important regulators of T cell responses. Short-chain fatty acids (SCFAs) produced by gut microbiota metabolism, by inhibiting HDAC and regulating the mTOR-S6K pathway, induce T cell effector and memory differentiation [313], representing host-internal microbiota-induced metabolic reprogramming. Moreover, a diet-derived trans-vaccenic acid (TVA) inactivates the cell surface receptor G-protein-coupled receptor 43 (GPR43)-an immune regulatory G-protein-coupled receptor activated by its short-chain fatty acid ligand [314]-thereby activating the cAMP-PKA-CREB axis, enhancing CD8^+^ T cell cytotoxic effector function which suppress tumor growth in melanoma mouse models, and representing host-extrinsic reprogramming of CD8^+^ T cells [315]. When the glucose availability of CD8^+^ T cells is low, a short-chain saturated fatty acid called acetate participates in the synthesis of acetyl-CoA under the catalysis of acetyl-CoA synthetase, thereby enhancing metabolism to maintain the supply of acetyl-CoA, which is an important intermediate product of energy metabolism, ultimately promoting CD8^+^ T cell proliferation and effector function in preclinical breast cancer models [316]. Intracellular fatty acids can also regulate T cell activation and proliferation by binding to PPAR [317]. However, the abundance of long-chain fatty acids (LCFAs) in the TME will cause T cell dysfunction, and regulating the expression of VLC acyl-CoA dehydrogenase (ACADVL) can improve the metabolic adaptability of TILs [318]. It has been found in mouses bearing melanoma that leptin-a lipid factor in fat metabolism-binds to the upregulated leptin receptor on TILs, induces phospho-p38 MAPK activation, further activates transcription factor ATF2, ultimately increasing TIL oxidative activity and ability [319]. Another lipid mediator, prostaglandin E 2 (PGE2) is metabolized by the enzyme from the twenty-carbon unsaturated fatty acid arachidonic acid. And its signal transduction with E-type prostanoid receptor 2/4 (EP_2_/EP_4_) is the key to inhibiting CD8^+^ T cell response to IL-2 signals, leading to the inability to expand the number of stem cell-like CD8^+^ T cells, and even causing cell ferroptosis due to mitochondrial dysfunction. Blocking the PGE2-EP_2_/EP_4_ axis achieves immune control over various solid tumors in mice [320, 321].

Physiological levels of cholesterol are necessary to maintain normal T cell function. A naturally occurring sterol, 7-alpha-hydroxycholesterol (7α-HC), exerts a significant inhibitory effect on the TCR signaling pathway by enhancing the membrane binding of CD3ε, thereby hindering its aggregation with LCK and suppressing LCK-mediated TCR phosphorylation. A brief pre-treatment of engineered T cells with 7α-HC can induce a reduction in their in vivo signaling, leading to an increase in the number and proportion of memory cells, thereby enhancing their long-term anti-tumor capabilities in mouse models of melanoma [322]. The enrichment of oxysterols in the TME changes the expression ratio of SREBP2/liver X receptor (LXR) [323, 324], which is the key transcription factor for intracellular cholesterol metabolism, leading to cholesterol deficiency in T cells, thereby causing CD8^+^ T cell metabolic programming to be silenced and enhancing its autophagy, resulting in increased CD8^+^ T cell dysfunction and apoptosis [325]. High cholesterol levels upregulate the expression of immune checkpoints on T cells, such as PD-1, 2B4, in an ER-stress-X-Box Binding Protein 1-dependent manner, thereby limiting CD8^+^ T_eff_ anti-tumor ability [326]. Cholesterol esters or cholesterol sulfate can comprehensively affect TCR signal transduction by changing TCR clustering or antigen affinity [327]. Therefore, regulating the types of fatty acids and cholesterol abundance in the TME is an effective way to regulate the anti-tumor ability of CD8^+^ T cells.

### Inorganic ion metabolism

Minerals in the TME, often in the form of messenger molecules or co-factors of biological enzymes, participate in signal transduction and metabolic regulation within T cells, thereby participating in T cell fate. The proliferation, maturation, and activation of T cells strongly depend on the activity of calcium release-activated channels (CRAC) activated by stromal interaction molecule 1 (STIM1) and calcium release-activated calcium modulator 1 (ORAI1) [328]. When CTLs are activated by antigens, the endoplasmic reticulum Ca^2+^ store is rapidly depleted, and the secretion of vesicles containing toxic cytokines is calcium-dependent [329]. At this time, STIM1/ORAI1 dependent Ca^2+^ entry is crucial for maintaining the ability of CD8^+^ T_eff_ to lyse tumor cells [330]. In addition, Store-operated Ca^2+^ entry guides CD8^+^ T cell metabolic reprogramming by activating NFAT and the PI3K-Akt kinase-mTOR nutrient-sensing pathway, supporting its anti-tumor response [331]. Mg^+^ can assist Ca^2+^ signal conduction. When T cells receive antigen stimulation, the expression of the magnesium transporter gene 1 (MAGT1) is upregulated, promoting Mg^+^ influx. The absence of MAGT1 causes a significant impairment of Ca^2+^ influx in T cells, leading to impaired response to antigen receptor binding [332]. Necrotic cells in tumors dissolve and release a large amount of K^+^, causing K^+^ accumulation in the TME. On the one hand, T cells perceive high extracellular K^+^ concentration and experience an obstacle to Akt-mTOR phosphorylation and effector programmes driven by TCR. Regulating the potassium channel Kv1.3 can improve the T cell dysfunction caused by this [333]. On the other hand, high extracellular K^+^ levels also inhibit T cell effector programs by limiting the uptake of nutrients, thereby inducing T cell autophagy, reducing the expression of exhaustion programs, and inducing CD8^+^ T cell stemness transformation and effector function inhibition [334]. Na^+^/K^+^ ATPase plays a key role in maintaining intracellular and extracellular K^+^ concentration. When the intracellular K^+^ level rises, it can prevent the destruction of reactive oxygen species (ROS), limit the exhausted state of T cells, and maintain their anti-tumor function, delay ultimately melanoma growth in mice [335]. Iron, as an important redox ion, participates in biochemical reactions within cells. When the activity of glutathione peroxidase 4 (GPX4) in cells decreases, lipid oxidation products cannot be metabolized by the glutathione reductase reaction catalyzed by GPX4, leading to a large accumulation of Fe^2+^ dependent lethal lipid ROS, eventually causing cell Ferroptosis [336]. It has been found that TILs are very sensitive to iron death, and GPX4 and ferroptosis suppressor protein 1 (FSP1) can enhance the iron death resistance of CD8^+^ TIL. In addition, a seizure risk-related protein DEP domain-containing protein5 (DEPDC5) protects CD8^+^ T cells from iron death and is necessary for CD8^+^ T cell-mediated anti-tumor immunity. Mechanistically, DEPDC5 defects cause an increase in mTORC1 activity in CD8^+^ T cells, thereby inducing ATF4 expression, and then increasing the expression of xanthine oxidase (XO) and lipid ROS, ultimately leading to spontaneous iron death [337]. Lithium salts, used clinically as mood stabilizers, have been found to have a role in regulating T cell metabolism [338]. Lithium carbonate has been confirmed to disrupt vacuolar ATPase, thereby halting lysosomal acidification and salvaging lysosomal diacylglycerol-PKCθ signaling, consequently promoting the localization of MCT1 to the mitochondrial inner membrane. This process further facilitates the entry of exogenous lactate into the mitochondria for oxidation, thereby improving the efficacy of T cell therapy for solid tumors in mouse [339].

## Conclusion and perspectives

With the advancements in tumor neoantigen identification, reactive T cell receptor (TCR) screening technologies, and gene engineering, alongside the profound exploration of structural immunology regarding the structure-function relationship of engineered T cells, TCR-engineered T cells have propelled the immunotherapy of refractory solid tumors into a new phase with superior specificity and stable functional activity. Concurrently, the recruitment of tumor antigen-specific soluble bispecific molecules facilitates and promotes the redirection of pre-existing polyclonal CD8^+^ T cells towards tumor cells, offering a simpler alternative for T cell therapy against solid tumors. However, exhaustion remains an intrinsic aspect of the anti-tumor fate of CD8^+^ T cells, rendering the mitigation or reversal of CD8^+^ T cell exhaustion imperative for ensuring sustained efficacy of anti-tumor immune therapy. Manipulating immune checkpoints enhances the adaptability of CD8^+^ T cells within tumors, while diverse cytokine engineering provides novel avenues for restoring CD8^+^ T cell activity. Various innovative approaches have been explored in CAR-T cell therapy for cancer. For instance, overexpression of *BCL-2* inhibits intrinsic apoptosis of T cells [340], overexpression of *FOXO1* regulates memory-like differentiation of T cells [341], preconditioning with appropriate temperature improves intratumoral movement of T cells [257], and drug-delivery biodegradable scaffolds that mimic the physiological microenvironment of T cell activation sustain prolonged T cell stimulation [342]. The success of these cases will provide practical evidence for TCR-engineered T cell therapy in solid tumors.

In summary, cell therapy based on highly antigen-sensitive, microenvironment-adaptive, and functionally complete T cells may currently be the most promising approach for the immunotherapy of solid tumors. In the future, the development of T cell therapy should focus on optimizing the design of engineered T cells and improving the efficiency of their industrial-scale production. By combinatorially engineering individual T cells to stably express tumor-specific high-affinity TCRs, obtain efficient co-stimulatory signals, and tolerate immune negative regulation, these cells will participate in the treatment of solid tumors with high environmental adaptability and low cross-reactivity. To this end, the application of systems biology approaches in transcriptomics, epigenomics, chromatin accessibility, and metabolomics, especially at the single-cell level, will continue to provide new insights for T cell therapy of solid tumors.

## Data Availability

Not applicable.

## Abbreviations

ACT: Adoptive cell transfer
ACADVL: VLC Acyl-CoA dehydrogenase
ADAM17: A disintegrin and metalloprotease 17
AMPK: AMP-activated protein kinase
AP-1: Activator protein 1
ATF: Activating transcription factor
BAF: BRG1 or BRM-associated factor
BATF: Basic leucine zipper ATF-like transcription factor
BAZ1B: Bromodomain adjacent to zinc finger domain, 1B
BCL10: B cell lymphoma/leukemia 10
BH4: Tetrahydrobiopterin
BiTE: Bispecific T cell engager
BTLA: B and T lymphocyte attenuator
cAMP: Cyclic adenosine monophosphate
CAR: Chimeric Antigen Receptor
CARD11: Caspase recruitment domain-containing protein 11
CARMA1: Caspase recruitment domain-containing membrane-associated guanylate kinase protein 1
CBL: Cbl proto-oncogene, E3 ubiquitin protein ligase
CBLB: Casitas B-lineage lymphoma proto-oncogene B
CD: Cluster of differentiation
CDR: Complementary determining region
CEA: Carcinoembryonic antigen
CEACAM1: Carcinoembryonic antigen-related cell adhesion molecule-1
CLDN: Claudin
Co-STAR: Co-stimulatory-STAR
CRAC: Calcium release-activated channels
CREB: cAMP- response element binding protein
CRISPR- Cas9: Clustered regularly interspaced short palindromic repeats- associated protein 9
CTL: Cytotoxic T lymphocytes
CTLA4: Cytotoxic T lymphocyte antigen 4
CYRIB: CYFIP related Rac1 interactor B
DAG: Diacylglycerol
DEPDC5: DEP domain-containing protein5
DLL3: Delta-like ligand 3
DSF: Disulfiram
EpCAM: Epithelial cell adhesion molecule
ERK: Extracellular signal-related kinase
FAM49B: The family with sequence similarity 49 member B
FAO: Fatty acid oxidation
FDA: Food and Drug Administration
Fgl2: Fibrinogen-like protein 2
FOXO1: Forkhead box O1
FSP1: Ferroptosis suppressor protein 1
GTP: Guanosine triphosphate
GCH1: GTP cyclohydrolase1
GLUT1: Glucose transporter 1
GOF: Gain-of-function
Got1: Glutamic-oxaloacetic transaminase 1
GPC3: Glypican-3
GPR43: G-protein-coupled receptor 43
GPX4: Glutathione peroxidase 4
GRB2: Growth factor receptor-bound protein 2
H3K27ac: Histone H3 Lysine 27 acetylation
HCC: Hepatocellular carcinoma
HDAC3: Histone deacetylase 3
HDR: Homology-directed repair
HIT: HLA-independent TCR
HLA: Human leukocyte antigen
HVEM: Herpesvirus entry mediator
ICB: Immune checkpoint blockade
Id2: Inhibitor of DNA binding protein 2
IFN: Interferon
iICPs: Inhibitory immune checkpoints
IKK: IĸB kinase
IL: Interleukin
ILRs: IL receptors
ImmTAC: Immune-mobilizing monoclonal TCR against cancer
INO80: Inositol requiring mutant 80
IP3: Inositol triphosphate
IRF4: Interferon regulatory factor 4
IRs: Inhibitory receptors
ITAMs: Immunoreceptor tyrosine-based activation motifs
ITIM: Immunoreceptor tyrosine-based inhibitory motif
ITPRIPL1: I-type single-pass membrane protein
ITSM: Immunoreceptor tyrosine-based activation motif
JAK: Janus kinase
KBs: Ketone bodies
LAG3: Lymphocyte activation gene-3
LAGE-1A: L antigen family member-1 A
LAT: linker for activation of T cells
LAT1: Large amino acid transporter 1
LCFAs: Long-chain fatty acids
LCMV: Lymphocytic choriomeningitis virus
LCP: Lymphocyte cytosolic protein
LDH: Lactate dehydrogenase
LNPs: Lipid nanoparticles
LSD1: Lysine demethylase 1
LXR: Liver X receptor
LCK: LCK proto-oncogene, Src family tyrosine kinase
MAGE-A4: Melanoma-associated antigen A4
MAGT1: Magnesium transporter gene 1
MALT1: Mucosa-associated lymphoid tissue translocation protein 1
MAPK: Mitogen-activated protein kinase
mCRPC: Metastatic castration-resistant prostate cancer
MCT1: Monocarboxylate transporter 1
MDSCs: Myeloid-derived suppressor cell populations
MEGA: Multiplexed effector guide arrays
MHC: Major histocompatibility complex
ModPoKI: Modular pooled knock-in screening
MPC: Mitochondrial pyruvate carrier
mRNA: Messenger ribonucleic acid
mSWI/SNF: Mammalians switch/sucrose non-fermentable
MTA: Methylthioadenosine
mTOR: Mammalian target of the rapamycin
mTORC: mTOR complex
MYC: MYC Proto-Oncogene
NeoTCR: Neoantigen-specific TCR
NFAT: Nuclear factor of activated T cells
NF-κB: Nuclear factor-ĸB
NGS: Next-generation sequencing
NY-ESO-1: New York esophageal squamous cell carcinoma 1
ORAI1: Calcium release-activated calcium modulator 1
Osr2: Odd-skipped related 2
PCK1: Phosphoenolpyruvate carboxykinase 1
PD-1: Programmed death protein-1
PDAC: Pancreatic ductal adenocarcinoma
PDK1: Phosphoinositide-dependent kinase 1
PGC-1α: PPAR gamma coactivator 1α
PGE2: Prostaglandin E 2
PIK3CD: Phosphatidylinositol-4,5-bisphosphate 3-kinase catalytic subunit delta
PIK3R3: Phosphoinositide-3-kinase regulatory subunit 3
PKC: Protein kinase C
p-MHC: Peptide- major histocompatibility complex
PPAR: Proliferator-activated receptor
PRAME: Preferentially expressed antigen in melanoma
PRODH2: Proline dehydrogenase 2
PSGL-1: P-selectin glycoprotein-1
PSIP1: PC4 and SFRS1 interacting protein 1
PSMA: Prostate-specific membrane antigen
PTKs: Protein tyrosine kinases
PTPN: Protein tyrosine phosphatases
RASA2: RAS P21 protein activator 2
RNP: Ribonucleoprotein
ROS: Reactive oxygen species
RC3H1: Ring finger and CCCH-type domains 1
SAM: S-adenosylmethionine
SCAP: SREBP cleavage-activating protein
SCFAs: Short-chain fatty acids
ScFv: Single-chain variable fragment
SCLC: Small-cell lung cancer
SERCA: Sarco/endoplasmic reticulum Ca^2+^-ATPase
SFXN1: Sideroflexin1
sgRNA: Single guide RNA
SHP: Src homology region 2 domain-containing phosphatase
SLICE: sgRNA lentiviral infection with Cas9 protein electroporation
SMAD4: SMAD family member 4
SNVs: Single-nucleotide variants
SOCS1: Suppressor of cytokine signaling 1
SREBP: Sterol regulatory element-binding transcription factor
SRs: Synthetic surface receptors
ssDNA: Single-stranded DNA
STAR: Synthetic TCR antigen receptor
STAT: Signal transducer and activator of transcription
STE-20: Serine/threonine-protein kinase 20
STIM1: Stromal Interaction Molecule 1
STS-1: Suppressor of T cell signaling-1
SynNotch: Synthetic Notch
TAOK3: Thousand-and- one–amino acid kinase 3
TCA: Tricarboxylic acid cycle
TCF: T-cell factor
Cα/Cβ: Constant domain α/β
TCR: T cell receptor
TCRm: TCRmimic
T_eff_: Effector T cell
T_ex_: Exhausted T cell
TF: Transcription factor
THEMIS: T-lineage protein thymocyte-expressed molecule involved in selection
TALENs: Transcription activator-like effector endonucleases
TIGIT: T cell immunoglobulin and ITIM domain
TILs: Tumor infiltrating lymphocytes
TMB: Tumor neoantigen mutation burden
TMD: Transmembrane domain
TME: Tumor microenvironment
TOX: Thymocyte selection-associated high mobility group box protein
TRAC: TCR constant domainα
TRBC: TCR constant domainβ
TSC: Tuberous sclerosis complex
TVA: Trans-vaccenic acid
UTRs: Untranslated regions
VISTA: V domain immunoglobulin suppressor of T cell activation
XO: Xanthine oxidase
ZAP: ζ-chain-associated protein
ZFNs: Zinc-finger nucleases
ZC3H12A: Zinc finger CCCH-type containing 12 A
7α-HC: 7-α-hydroxycholesterol
